# Genomic potential and physiological characteristics of C1 metabolism in novel acetogenic bacteria

**DOI:** 10.3389/fmicb.2023.1279544

**Published:** 2023-10-19

**Authors:** Jihyun Yu, Mi-Jeong Park, Joungmin Lee, Soo Jae Kwon, Jae Kyu Lim, Hyun Sook Lee, Sung Gyun Kang, Jung-Hyun Lee, Kae Kyoung Kwon, Yun Jae Kim

**Affiliations:** ^1^Korea Institute of Ocean Science and Technology, Busan, Republic of Korea; ^2^KIOST School, University of Science and Technology, Daejeon, Republic of Korea

**Keywords:** acetogenic bacteria, C1 compounds, formate, carbon monoxide, methanol, ES strains

## Abstract

Acetogenic bacteria can utilize C1 compounds, such as carbon monoxide (CO), formate, and methanol, via the Wood-Ljungdahl pathway (WLP) to produce biofuels and biochemicals. Two novel acetogenic bacteria of the family *Eubacteriaceae* ES2 and ES3 were isolated from Eulsukdo, a delta island in South Korea. We conducted whole genome sequencing of the ES strains and comparative genome analysis on the core clusters of WLP with *Acetobacterium woodii* DSM1030^T^ and *Eubacterium limosum* ATCC8486^T^. The methyl-branch cluster included a formate transporter and duplicates or triplicates copies of the *fhs* gene, which encodes formyl-tetrahydrofolate synthetase. The formate dehydrogenase cluster did not include the hydrogenase gene, which might be replaced by a functional complex with a separate electron bifurcating hydrogenase (HytABCDE). Additionally, duplicated copies of the *acsB* gene, encoding acetyl-CoA synthase, are located within or close to the carbonyl-branch cluster. The serum bottle culture showed that ES strains can utilize a diverse range of C1 compounds, including CO, formate, and methanol, as well as CO_2_. Notably, ES2 exhibited remarkable resistance to high concentrations of C1 substrates, such as 100% CO (200 kPa), 700 mM formate, and 500 mM methanol. Moreover, ES2 demonstrated remarkable growth rates under 50% CO (0.45 h^−1^) and 200 mM formate (0.34 h^−1^). These growth rates are comparable to or surpassing those previously reported in other acetogenic bacteria. Our study introduces novel acetogenic ES strains and describes their genetic and physiological characteristics, which can be utilized in C1-based biomanufacturing.

## 1. Introduction

One carbon (C1) compounds, such as carbon dioxide (CO_2_), carbon monoxide (CO), formate, and methanol, have recently gained attention as sustainable non-food and low-cost alternative feedstocks for industrial biomanufacturing (Müller, 2019; Cotton et al., 2020; Lv et al., 2022). Formate can be synthesized by the electrochemical reduction of CO_2_ or obtained through partial oxidation of natural gas or syngas (Yishai et al., 2016). CO is a major component of syngas generated by coal or biomass gasification, and its biological conversion into chemicals has been extensively investigated in acetogenic bacteria (Köpke et al., 2011). In particular, *Clostridium autoethanogenum* has shown promise for commercial-scale ethanol production from syngas (Liew et al., 2016). Methyl group-containing C1 compounds include methane and methanol. Methanol is the simplest type of alcohol and can be derived by the simple conversion of methane, which naturally exists in vast quantities in natural gas. However, it can also be produced by hydrogenation of CO_2_ using hydrogen gas (H_2_) (Chen et al., 2019; Wiesberg et al., 2019). Biological conversion of these C1 compounds to biofuels or biochemicals has been considered a sustainable approach for replacing fossil resources (Müller, 2019; Cotton et al., 2020; Kremp and Müller, 2021).

**Figure 1:**
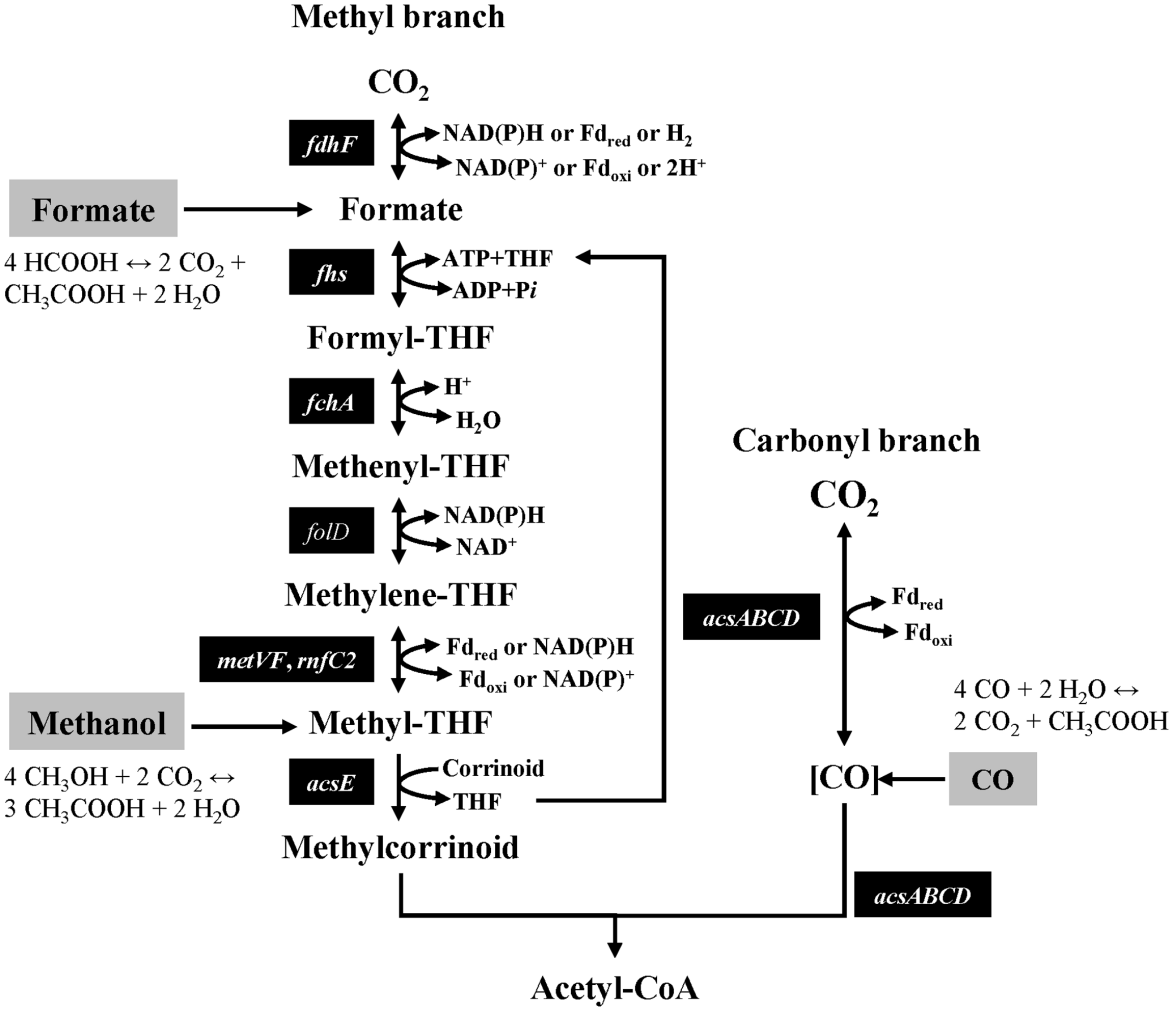
Schematic model of the WLP and incorporation of C1 compounds as intermediates.

Acetogenic bacteria are obligate anaerobes that can use the Wood-Ljungdahl pathway (WLP) for the assimilation of CO_2_ to produce cellular carbon (Figure 1). These acetogenic bacteria are phylogenetically diverse and have been assigned to 23 genera, and more than 100 species have been identified from various anoxic environments, including some extreme habitats (Denned, 2006; Wiechmann and Müller, 2019). The WLP involves fewer genes than other CO_2_ fixation pathways and is highly energy efficient (Schuchmann and Müller, 2014; Claassens et al., 2019). Interestingly, WLP contains pathways that can utilize various C1 compounds, such as CO, formate, and methanol, as intermediates for carbon or energy sources in addition to CO_2_ (Figure 1).

Formate provides not only the carbon of the methyl group in acetyl-CoA but also reduces equivalents that are required for carbon fixation in WLP, thereby enabling the autotrophic growth of several acetogens. From each 4 mol of formate, 3 mol are oxidized to CO_2_ to provide the reducing power required to combine one formate and one CO_2_ into one acetate, ultimately yielding 2 mol of CO_2_ (Figure 1). Some acetogenic bacteria, including *Acetobacterium woodii* and *Eubacterium limosum* can use formate as a carbon and energy source (Moon et al., 2021; Wood et al., 2022). The mechanism of formate metabolism has been clearly elucidated in the case of *A. woodii*, it is known that the key enzyme is formate dehydrogenase (FDH), which acts as a CO_2_ reductase at the methyl branch in WLP. FDHs in acetogenic bacteria have been classified according to the specific electron donor used and the metal cofactor in the active site. For instance, thermophilic acetogen *Moorella thermoacetica* has a tungsten-selenium-containing NADPH-specific FDH (Andreesen and Ljungdahl, 1973; Yamamoto et al., 1983; Deaton et al., 1987). Moreover, the primary structure of FDH of *C. autoethanogenum* forms a functional complex with electron bifurcating hydrogenase specific to NADPH (Wang et al., 2013; Schuchmann and Müller, 2014). The FDH/hydrogenase complex has also been reported in *E. callanderi* KIST612. In contrast to *Clostridium* spp., it requires ferredoxin and NADH as electron carriers and shows a strong bias to formate oxidation reactions (Dietrich et al., 2021). A hydrogen-dependent CO_2_ reductase (HDCR) was found in *A. woodii* and *Thermoanaerobacter kivui*, which has the highest biohydrogen production rate known (Schuchmann and Müller, 2013; Schwarz et al., 2018; Burger et al., 2022). *A. woodii* showed the highest growth rate of 0.12 h^−1^ with formate, with one strain that can withstand a concentration of up to 0.5 M (Moon et al., 2021). Specifically, it has been reported that *T. kivui* can completely restore its growth rate by using formate instead of CO_2_ in mannitol metabolism (Moon et al., 2020). The growth rate of known mesophilic acetogenic bacteria was reported to be in the range of 0.03–0.12 h^−1^ (Cotton et al., 2020).

Additionally, as an intermediate of the carbonyl branch in the WLP, CO can either be directly assimilated to acetyl-CoA or oxidized to CO_2_ to provide a reducing equivalent for cell growth. A key enzyme is carbon monoxide dehydrogenase (CODH), which reduces CO_2_ to CO by forming a complex with acetyl-CoA synthase (ACS) at the carbonyl branch in the WLP. Genes encoding codh and acs are clustered together, and most of them are well-conserved in acetogenic bacteria (Müller, 2003). Some acetogenic bacteria, such as *C. autoethanogenum*, *C. ljungdahlii*, *Clostridium sp. AWRP*, *A. bakii*, *E. limosum*, *E. callanderi*, and *Thermoanaerobacter kivui*, can grow using CO as a carbon and energy source (Tanner et al., 1993; Abrini et al., 1994; Chang et al., 1998; Sattley and Madigan, 2007; Weghoff and Müller, 2016; Lee et al., 2019; Kang et al., 2020). Hydrogenases are inhibited in the presence of CO; however, as mentioned earlier, the types involved in the WLP vary among different strains. Consequently, each strain exhibits distinct levels of resistance to CO, directly impacting their growth rates. The acetogen with the highest growth rate under CO conditions was *E. callanderi* at 0.17–0.25 h^−1^ in a gas-lift reactor, and other acetogenic bacteria exhibited growth rates within the range of 0.04–0.16 h^−1^ (Chang et al., 1998; Cotton et al., 2020).

As an intermediate of the methyl branch in the WLP, the methyl group (methanol) is transferred by a methyltransferase system to tetrahydrofolate (THF) in the methyl branch. Of the 4 mol of methyl-THF, 1 mol is completely oxidized to CO_2_ to provide the reducing power required to reduce the generated CO_2_ and an additional 2 mol of CO_2_ to 3 mol CO (Figure 1). Some acetogenic bacteria, including *E. limosum* and *A. woodii*, can grow using methanol as a carbon and energy source. Recently, mta genes related to methanol metabolism were identified in *A. woodii*, and the biochemical characteristics of these enzymes were identified (Kremp et al., 2018). Methanol metabolism in acetogenic bacteria is more advantageous than aerobic methylotrophic pathways because it bypasses the production of reactive intermediate product formaldehyde, which largely contributes to the toxicity of methanol (Tremblay et al., 2015; Müller, 2019). Most acetogens cannot tolerate methanol at concentrations above 100 mM, but strains subjected to laboratory evolution have been reported to be able to grow at high methanol concentrations (~ 0.5 M) (Tremblay et al., 2015). Additionally, the species with the highest growth rate under methanol conditions was *E. limosum* at 0.11 h^−1^, and the growth rate of known acetogenic bacteria was reported to be in the range of 0.07–0.11 h^−1^ (Cotton et al., 2020).

Formate and methanol are promising C1 substrates in terms of energy efficiency, with advantages in mass transfer due to its water-soluble character, but disadvantages in terms of product yields (Claassens et al., 2019; Cotton et al., 2020). In contrast, C1 gasses (H_2_, CO) have challenges associated with complicated reactors, storage and distribution of the gasses (Yasin et al., 2015; Yishai et al., 2016; Cotton et al., 2020). The potential of acetogenic bacteria in the industrial conversion of C1 compounds is very promising. However, they are restrained by a narrow product spectrum and relatively low growth rates depending on the substrate. Therefore, to develop industrially applicable acetogenic strains, it is imperative to discover novel species possessing multipotential for C1 compound metabolism. Here, we addressed novel acetogenic bacteria, ES strains, and their characteristics and genomic potential for C1 metabolism.

## 2. Materials and methods

### 2.1. Sample collection and isolation

Sediment core samples were collected from the triangular site in Eulsukdo Island in Busan, South Korea (35°06′35.0″N, 128°56′46.0″ E). Core samples from a depth of 12–24 cm from the surface were divided into 3–4 cm lengths. Each sample was dissolved to form sludge and inoculated in an enrichment culture medium. We prepared four types of autotrophic medium that employed H_2_:CO_2_ (8:2, v/v) gas as a substrate to stimulate the growth of acetogenic bacteria that displayed chemolithoautotrophic growth. Among the enrichment samples, sediment at a depth of 4–8 cm from the Eulsukdo region showed substrate consumption, and four strains were isolated as pure cultures. The medium composition used for the isolation of ES strains was made based on the German Collection of Microorganisms and Cell Cultures (DSMZ, Germany) 711a medium. Each liter of the modified 711a medium contained NH_4_Cl 1.00 g, K_2_HPO_4_ 0.30 g, KH_2_PO_4_ 0.30 g, MgCl_2_ x 6 H_2_O 0.20 g, CaCl_2_ x 2 H_2_O 0.10 g, KCl 0.10 g, NaCl 15.00 g, yeast extract 0.05 g, Na-acetate x 3 H_2_O 0.50 g, Na-resazurin solution (0.1% w/v) 0.50 mL, Na_2_CO_3_ 1.50 g, l-Cysteine-HCl x H_2_O 0.30 g, Na_2_S x 9 H_2_O 0.30 g, vitamin solution (refer to DSMZ medium 141) 10.00 mL, trace element solution SL-10 (refer to DSMZ medium 320) 1.50 mL, and distilled water 1,000.00 mL.

### 2.2. Strains and growth conditions

To validate the taxonomic position of the isolated strains, the 16S rRNA-based phylogenetic tree and a distance matrix were constructed. The 16S rRNA sequences of 15 closely related species belonging to the genera *Eubacterium* and *Acetobacterium* were obtained from the NCBI database. Additionally, the 16S rRNA sequences of the type species of the genera *Alkalibaculum* and *Alkalibacter* were used as an outgroup in the phylogenetic analysis. The phylogenetic tree was constructed via the neighbor-joining method using the Jukes and Kantor distance model and the maximum likelihood and maximum parsimony method with 1,000 bootstrap values using MEGA version 7.0 (Jukes and Cantor, 1969; Fitch, 1971; Felsenstein, 1981; Saitou and Nei, 1987; Tamura et al., 2004; Kumar et al., 2016). Growth experiments were conducted to determine the optimal NaCl concentration, pH, and temperature for ES strains by recording the optical density at 600 nm every 10 min for 72 h through a temperature gradient incubator (TVS126MA, Adaventec). Temperatures from 15–45 (15, 20, 22.6, 25.4, 27.7, 30, 32.3, 35, 37, 39.3, 42.1, 45) °C, NaCl concentrations from 0–5.5 (0, 0.5, 1, 1.5, 2, 2.5, 3, 3.5, 4, 4.5, 5, 5.5) %, and pH values from 4–9.7 (4, 4.5, 5, 5.5, 5.9, 6.3, 6.8, 7.3, 7.8, 8.3, 8.8, 9.3, 9.7) were used. The modified DSMZ 711a medium in this experiment was supplemented with 20 mM of citrate phosphate buffer (for pH 4.0–5.5), 20 mM of MES (for pH 5.5–6.8), 20 mM of HEPES (for pH 6.8–8.3), or 20 mM of AMPSO (pH 8.3–9.7).

### 2.3. Genomic analysis and calculation of genomic indices

For genomic DNA sequencing, a PacBio 20 kb library was prepared and sequencing analysis was performed in Chunlab (Seoul, South Korea). PacBio Sequencing data was assembled using PacBio RSII SMRT Analysis 2.3.0 with HGAP2 protocol (Pacific Biosciences, USA). For the accurate taxogenomic analysis of the sequenced genomes, the genomes of the selected strains for 16S rRNA-based phylogeny were obtained from the publicly available NCBI GenBank database. Genome annotation was performed using Prokka v1.14.6 to identify and annotate various genetic features (Seemann, 2014). For phylogenomic analysis, we utilized the PhyloPhlan v3.0.67 pipeline (Asnicar et al., 2020). First, 400 marker proteins were extracted from the annotated genomes in fasta format. Second, multiple sequence alignment of the marker proteins was carried out using MAFFT v7.520 (Katoh and Standley, 2013). Third, tree reconstruction was conducted by generating an initial phylogenetic tree using FastTree v2.1.11 and then refining and optimizing the tree topology using RAxML v8.2.12 (Price et al., 2009; Stamatakis, 2014). For comparison of genomic indices, four metrics were calculated, namely average nucleotide identity (ANI), *digital* DNA–DNA hybridization (*d*DDH), average amino acid identity (AAI), and percentage of conserved proteins (POCP). To determine ANI for species delineation, we used the OrthoANI-usearch tool version 1.2 (OAUv1.2) (Yoon et al., 2017). For *d*DDH, we obtained the values using genome-to-genome distance calculator version 3.0, a web service provided by DSMZ (Meier-Kolthoff et al., 2013). For genus delineation, AAI was calculated using the AAI calculator, a web service provided by the Kostas lab. Additionally, POCP was calculated using a Python script based on the method described in a previous study (Qin et al., 2014).

### 2.4. Comparative genomics

Gene finding and functional annotation were performed using the following procedure. Protein-coding sequences (CDSs) were predicted via Prodigal v 2.6.2 for rRNA by INFERNAL version 1.0.2 and for tRNA by tRNA-scan version 1.3.1. The UBLAST algorithm was used for the functional annotation of the predicted CDSs with reference to the EggNOG, SwissProt, KEGG, and SEED databases and complemented by the NCBI Prokaryotic Genome Annotation Pipeline. The orthologous gene clusters of the ES strains of *A. woodii* DSM 1030^T^ and *E.limosum* ATCC 8486^T^ were analyzed by OrthoVenn2 web server^1^ to identify protein clusters of orthologs or paralogs from comparative strains. The E-value cutoff was set at 0.05 for protein similarity comparison, and a 1.5 inflation value was applied to the Markov Cluster Algorithm (Van Dongen, 2000).

### 2.5. C1 compound utilization

To test the growth potential and metabolite of ES strains on a variety of C1 compounds, ES strains of *A. woodii* DSM 1030^T^ and *E.limosum* ATCC 8486^T^ were grown under each optimal condition with a variety of C1 compounds as substrates. The C1 compounds used in the experiment were H_2_/CO_2_, CO, formate, methanol, and formaldehyde. The gaseous C1 compounds H_2_/CO_2_ (80:20, v/v) and 20% of CO (CO/N_2_, 20:80, v/v) were supplemented at a pressure of 200 kPa and 125 kPa, respectively. The miscible C1 compounds formate, methanol, and formaldehyde were supplemented at a concentration of 60 mM. After 1 to 2 weeks of incubation, substrate consumption, and growth metabolites were analyzed using gas chromatography (YL 6100; YL Instrument Co., Anyang, South Korea) and HPLC-RID system (YL9100; YL Instrument Co.) furnished with an Aminex HPX-87H (300 × 7.8 mm; Bio-Rad, CA) column using sulfuric acid (5 mM) as the mobile phase.

## 3. Results and discussion

### 3.1. Initiative phylogeny and phenotypic characteristics of the novel ES strains

**Figure 2:**
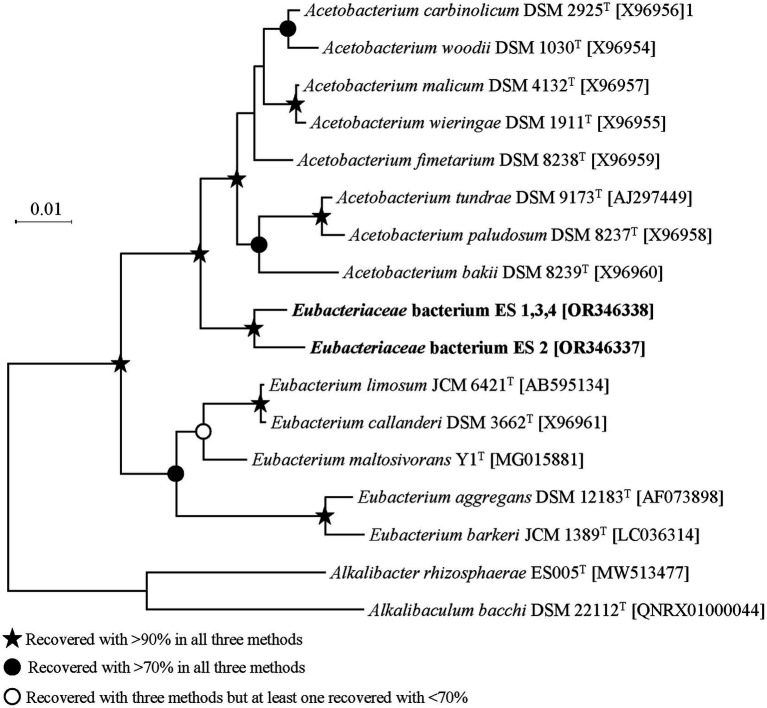
Phylogenetic tree of ES strains based on aligned 16S rRNA gene. The symbols of branches indicate the percentage of data coverage for the internal node by three methods (neighbor-joining, maximum likelihood, and maximum parsimony) with 1,000 bootstraps.

ES strains were isolated from the same sediment sample, but some strains exhibited physiologically different properties. The 16S rRNA sequence-based phylogeny results and distance matrix showed that ES1, ES3, and ES4 belonged to the same species. Therefore, for the subsequent analysis, our focus will be on ES2 and ES3, as they represent distinct species based on the phylogenetic analysis (Figure 2). Both ES strains were mesophilic, showing optimal growth at 30–35°C but had different optimum pHs and salinities for growth. ES2 exhibited optimal growth at pH 7.8 and 0.5% of NaCl, whereas ES3 required a pH of 6.8 and 2.5% NaCl (Supplementary Table S1).

16S rRNA sequence homology showed that ES2 and ES3 had 97.9% similarity and were most closely related to *Acetobacterium fimetarium* (96.8 and 97.0%, respectively). Within the phylogenetic tree ES strains were clustered in coherent branches and were phylogenetically located between the genera *Acetobacterium* and *Eubacterium*. The general genomic features of ES strains are detailed in Supplementary Table S2. The genome sizes of ES2 and ES3 were 3.76 Mbp and 3.39 Mbp, respectively. The genomes of ES2 and ES3 consisted of single circular DNA with G + C contents of 40.7 mol% and 39.6 mol% and with 3,619 and 3,392 protein-coding genes, respectively. The phylogenomic comparison with related species exhibited a higher and more reliable bootstrap value than the 16S rRNA-based phylogenetic tree, bolstering the overall reliability of the analysis. ES2 and ES3 formed a distinct cluster separate from the *Eubacterium* spp. However, whether ES2 and ES3 are distinct from the genus *Acetobacterium* required further analysis (Supplementary Figure S1). Therefore, species and genus delineation was carried out by comparing genomic indices. The results of ANI and *d*DDH comparisons confirmed that ES2 and ES3 are novel entities at the species level (Supplementary Figure S2). The AAI was then examined to determine novelty even at the genus level. When comparing the genomes of ES2 and ES3 with those of the genus *Acetobacterium*, a maximum homology of 66.9 was found, while the genomes within the genus *Acetobacterium* exhibited a homology of 72 or more (Supplementary Figure S3). Similarly, when compared with the genus *Eubacterium*, ES2 and ES3 showed a maximum homology of 56.3, indicating their distinction from the genus. A rigorous comparison of phylogeny and genomic indices at the genome level indicated that ES2 and ES3 exhibit novelty not only at the species level but also at the genus level.

### 3.2. Comparative genomic analysis of ES strains

In this study, we investigated the conservation of the WLP genes in the genomes of the ES strains and predicted their metabolites. To gain insight into the genetic characteristics of these novel acetogenic ES strains, we performed a comparative genomic analysis with the taxonomically related acetogen species *A. woodii* and *E. limosum*. Synteny analysis revealed that, in terms of genome arrangement, the ES strains were closer to *A. woodii* than to *E. limosum* (Supplementary Figure S4). The analysis of orthologous gene clusters showed that 1,344 clusters, composed of 5,574 proteins including key enzymes of WLP, were shared between the comparative and ES strains (Supplementary Figure S5). The core molecular functions occupying the majority of shared clusters included ion binding GO:0043167, hydrolase activity GO:0016787, and oxidoreductase activity GO:0016491. Among the 2,573 clusters of ES strains, 418 were not shared between the two comparison strains, and among these, 50 and 29 unique clusters were found in ES2 and ES3, respectively (Supplementary Figure S5). Clusters shared by ES strains and *A. woodii* amounted to 1,900, whereas they numbered 1,419 with *E. limosum*. This observation confirms that the gene cluster composition of ES strains is also more similar to *A. woodii* than to *E. limosum*.

### 3.3. Formate metabolism of ES strains

**Figure 3:**
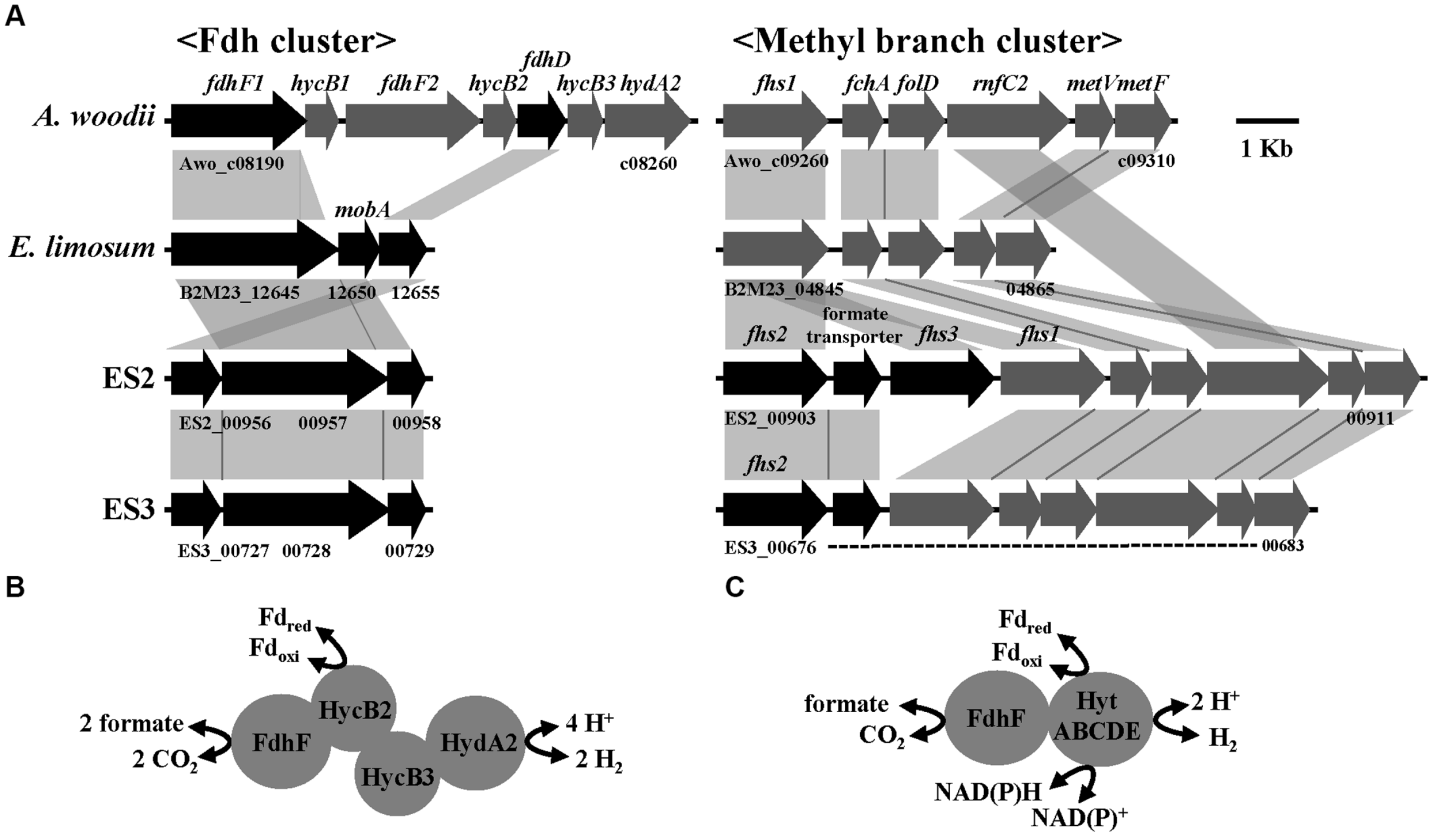
Formate metabolism in ES strains. **(A)** Comparison of gene organization in formate dehydrogenase and methyl branch cluster of four strains. **(B)** Model of HDCR reported in *Acetobacterium woodii*. **(C)** Predicted functional complex of FDH and bifurcating hydrogenase in ES strains.

Formate metabolism is well-characterized in the acetogen *A. woodii* and is closely related to the WLP (Moon et al., 2021). In *A. woodii*, formate is introduced downstream of WLP and used as a precursor to form the methyl branch of acetyl-CoA. Conversely, it can also be introduced upstream of WLP, wherein it is decomposed into H_2_ and CO_2_ by HDCR, enabling the reduction of CO_2_ to CO (Moon et al., 2021). One of the key enzymes in formate metabolism is FDH, which catalyzes the oxidation of formate to produce CO_2_ and H_2_ (Figures 1, 3A). The *fdh* cluster of *A. woodii* comprised two modular structures, including formate dehydrogenase and hydrogenase, and the gene of the former was present in two copies, one with selenium-containing (*fdhF2*) and the other without (*fdhF1*) (Schuchmann and Müller, 2013). Recent studies in physiology, biochemistry, and bioenergetics have well elucidated the function and role of HDCR (Schuchmann et al., 2016). On the other hand, the FDH of *Clostridium autoethanogenum* forms a functional complex (FDH-HytABCDEE2) with electron-bifurcating [FeFe] hydrogenase, which performs the same role as HDCR (Schuchmann and Müller, 2013; Wang et al., 2013; Kottenhahn et al., 2018; Lee et al., 2022). Such a functional complex has also been observed in *E. limosum* ATCC 8486^T^ and *E. callanderi* DSM 3662^T^ (Song et al., 2017). However, unlike in *Clostridium* spp., the genes encoding FDH and hydrogenase are separated into two distinct clusters, and the hydrogenase is composed of five genes (HytABCDE), which closely resemble the gene organization in the ES strains (Figures 3A,C; Dietrich et al., 2021). To analyze the predicted formate metabolic pathways, the *fdh* and methyl branch gene clusters of *A. woodii, E. limosum*, and ES strains were compared (Figure 3). As shown in Figure 3B, the HDCR of *A. woodii* comprises a formate dehydrogenase module and a [FeFe] hydrogenase module connected by two electron-transferring subunits (Schuchmann and Müller, 2013). In contrast, the hydrogenase encoding genes were absent in the ES strains, and the formate dehydrogenase cluster, including the *fdh*, *mob*, and *fdh*D encoding genes, was organized in a similar way to *E. limosum* (Figure 3A), The FDH from ES2 and ES3 strains showed the highest homology to that of *Acetobacterium wieringae* (88%). According to the recently reported data, the *fdh* variants in *Acetobacterium* spp. formed four distinct clusters: one *A. woodii* type *fdh* cluster (I) and three non-*A. woodii* type *fdh* clusters (IA, IB, and IC) (Ross et al., 2020). The *fdh* variant of the ES strains was consistent with cluster IC. Furthermore, An N-terminal extension of FDHs from ES2, ES3, and *E. limosum* is present in contrast to that of *A. woodii*, and this extension contains conserved domains for a [2Fe_2_S] and two [4Fe_4_S] clusters, which are also present in NAD(P)H-dependent FDHs (Hartmann et al., 2015). The predicted FDH does not contain a NAD(P)H-binding site and is related to Fd-dependent enzymes (Liu and Mortenson, 1984) as well as a molybdopterin oxidoreductase involved in H_2_ oxidation in *Desulfovibrio desulfuricans* G20 (Li et al., 2009). In the methyl branch, related genes were well conserved in all strains except for the *rnfC2* gene of *E. limosum*. Genome mining of *A. woodii* revealed two genes encoding potential formyl-THF synthetase (*fhs*). One gene is located at the methyl branch cluster of WLP and the other one forms an operon with a gene encoding a predicted formate transporter (*fdhC*) (Müller, 2019; Moon et al., 2021). Interestingly, unlike *A. woodii*, the gene encoding FHS in ES2 and ES3, was triplicated and duplicated, respectively, and located in the same cluster (Figure 3A). This feature was observed in the methyl branch clusters from *A. paludosum* and *A. tundra* and might be present as an adaptive trait (Esposito et al., 2019). In addition, the formate transporter genes from the ES strains were in the same cluster. The genes involved in formate metabolism were probably clustered together so that they could work efficiently. These genomic features are expected to provide valuable insights into the mechanism behind the different phenotypes in formate metabolism in ES strains, *A. woodii*, and *E. limosum*.

**Figure 4:**
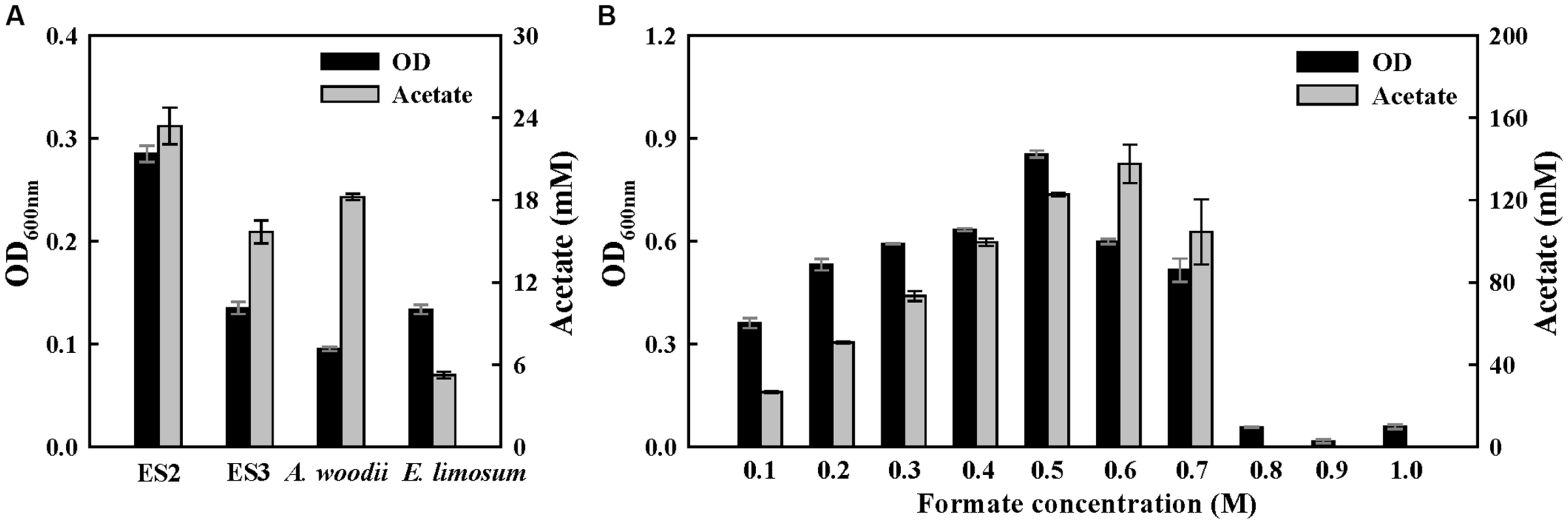
Growth of ES stains on formate. **(A)** Final optical densities and product concentration after a 1-week batch culture on 60 mM sodium formate. **(B)** Growth effect of formate concentration (100 mM to 1 M) in ES2. All experiments were performed in duplicate.

To confirm the potential of formatotrophic growth, the four strains were cultured for 1 week in a medium supplemented with 60 mM formate. All strains were able to grow with formate as a carbon and energy source and produced acetate as the main product (Figure 4A). Among them, the ES2 strain completely consumed supplemented formate and exhibited the highest optical density (OD) (0.28) and acetate production (23.4 mM). While ES3 strain showed a slightly higher OD than *A. woodii*, but lower acetate production. Moreover, *A. woodii* completely consumed the formate whereas ES3 had 4.5 mM remaining. To examine the effect of formate concentrations on the growth of ES2, a growth test was performed at formate concentrations of 0.1–1.0 M. The ES2 strain was able to grow at a formate concentration of 0.7 M, and the final OD and acetate production were the highest at 0.5 M and 0.6 M, respectively (Figure 4B). However, the growth rate (0.34 h^−1^) was the highest at 0.2 M formate concentration (Supplementary Figure S6). Compared to ES2, *A. woodii* was less resistant to formate with a lower maximum concentration (0.5 M) and growth rate (0.12 h^−1^) (Moon et al., 2021). Interestingly, the growth rate of ES2 in the presence of formate (0.34 h^−1^) was the highest among acetogens, exceeding the reported range of 0.03–0.08 h^−1^ (Cotton et al., 2020), and similar to that of the aerobic bacterium *Ralstonia eutropha* (0.18 h^−1^), which uses the Calvin cycle for formate metabolism (Grunwald et al., 2015). However, the energetic efficiency of bioproduction during formate metabolism via the Calvin cycle (25–35%) was significantly lower than that via the WLP (89%) (Cotton et al., 2020).

### 3.4. CO metabolism of ES strains

**Figure 5:**
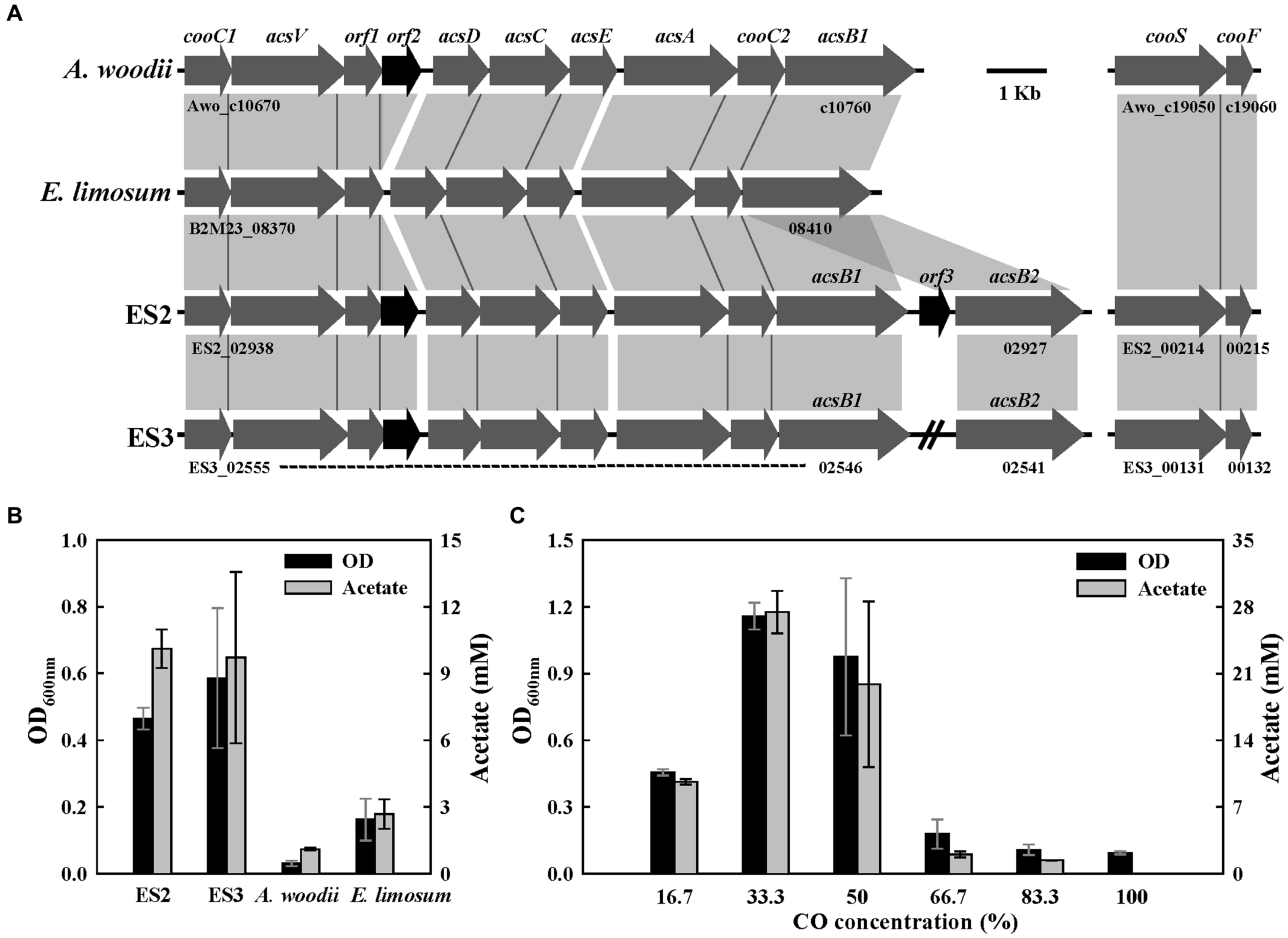
CO metabolism in ES strains. **(A)** Comparison of gene organization in carbonyl branch cluster of four strains. **(B)** Final optical densities and product concentration after 1 week of batch culture under 20% of CO gas (N_2_/CO, 8:2, 125 kPa). **(C)** Effect of CO concentration on growth (16.7–100%, remaining gas composition being N_2_, 150 kPa) in ES2. All experiments were performed in duplicate.

CO metabolism in the acetogen *E. limosum* is well-characterized and is closely related to the WL pathway (Drake et al., 2008; Kang et al., 2020). To analyze the predicted CO metabolic pathways, the carbonyl branch gene clusters of four strains were compared. The carbonyl branch gene cluster encodes the methyltransferase (AcsE), which converts methyl-THF to methylcorrinoid, and the CO dehydrogenase/acetyl-CoA synthase complex (AcsABCD) with the accessory proteins (CooC and AcsV), which convert methylcorrinoid and CO to acetyl-CoA (Figures 1, 5A). Unlike the *fdh* and methyl branch clusters, the carbonyl branch gene cluster was well conserved in the four strain genomes, except for the orf2 (hypothetical protein), which was absent from the genome of *E. limosum* (Figure 5A). Furthermore, all strains except *E. limosum* contained additional monofunctional CO dehydrogenase genes (*cooS* and *cooF*) that were in a separate cluster from the carbonyl branch gene cluster. In particular, in the ES strains genome, another copy of the *acsB* gene, encoding acetyl-CoA synthase, was present within or near the cluster (Figure 5A). This characteristic has not been previously reported in acetogens, and similar to gene duplication, it may also be a present as an adaptive trait. These genomic features are expected to represent the characteristics of different phenotypes in the CO metabolism of ES strains, *A. woodii*, and *E. limosum*.

To confirm the potential of carboxydotrophic growth, the above four strains were cultured for 1 week in a medium supplemented with 20% CO. All strains except *A. woodii* were able to grow on CO as a carbon and energy source and produced acetate as the main product (Figure 5B). ES strains showed high growth and acetate production compared to *E. limosum* (Figure 5B). Previous studies have proposed that the presence of additional monofunctional CODH (*cooS*) may influence the efficiency of CO metabolism (Weghoff and Müller, 2016). The growth experiment results shown in Figure 5 indicate that *E. limosum* might not efficiently utilize CO, possibly due to the absence of the *cooS* gene. According to a previous study, native *A. woodii* is unable to utilize CO as a sole carbon and energy source (Bertsch and Müller, 2015). Despite the similarity in genomic content, the ES strains but not *A. woodii* grow under CO conditions due to the difference in the FDH/hydrogenase complex involved in converting CO_2_ to formate in the methyl branch. The HDCR of *A. woodii* is sensitively inhibited in the presence of CO and incapable of producing formate. This is supported by evidence that the inhibition of *A. woodii* growth by CO is alleviated by the addition of formate (Bertsch and Müller, 2015). In contrast, the FDH/hydrogenase complex of the ES strains demonstrates resistance to CO. To examine the effect of CO concentrations on the growth of ES2, a growth test was performed at 17–100% CO concentrations (with the remaining gas being N_2_, at a total pressure of 150 kPa). ES2 showed the highest OD (1.16) and acetate concentration (27.4 mM) with CO at a concentration of 33% and could grow with concentrations reaching 50%, but failed to grow with concentrations above 67% (Figure 5C). Subsequently, through several generations of subculturing, the resistance of ES2 to high CO concentrations was improved, making it capable of growing with 100% CO (Supplementary Figure S7). During the growth of ES2 with CO, the only observed products were acetate and CO_2_, with a product recovery rate of over 99%. In this study, ES2 exhibited a remarkable growth rate of 0.45 h^−1^ under the conditions of 50% CO, 10% CO_2_, 10% H_2_, and 30% N_2_ (100 kPa) (Supplementary Figure S6). *E. callanderi* KIST612, which forms an FDH/hydrogenase complex similar to that predicted in the ES strains, also exhibits high resistance to CO and demonstrates a rapid growth rate with CO (0.17–0.25 h^−1^) (Chang et al., 1998). The growth rate of adaptively evolved *E. limosum* ATCC 8486 T was reported to reach 0.095 h^−1^ (44% CO/22% CO_2_/2% H_2_, 200 kPa) (Kang et al., 2020). In contrast, *C. autoethanogenum* showed a growth rate of 0.057 h^−1^ (45% CO/20% CO_2_/2% H_2_, 200 kPa) (Marcellin et al., 2016). The growth rate of ES2 (0.45 h^−1^) was the highest among acetogenic bacteria under carboxydotrophic conditions, surpassing the previously reported range of growth rates (Cotton et al., 2020).

### 3.5. Methanol metabolism of ES2 strain

**Figure 6:**
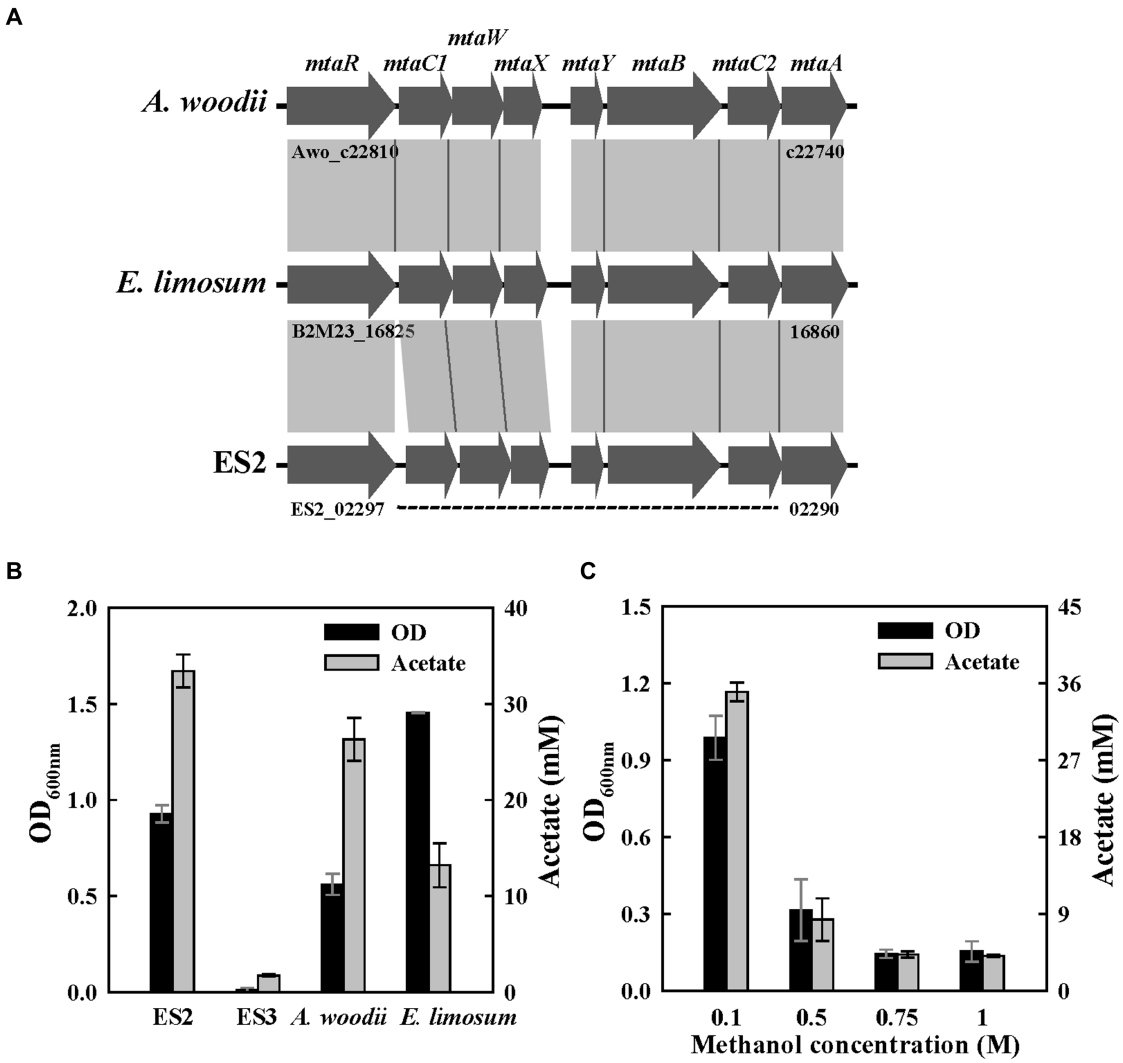
Methanol metabolism in ES strains. **(A)** Comparison of gene organization in methyltransferase cluster of three strains. **(B)** Final optical densities and product concentration after a 1-week batch culture on 60 mM of methanol. **(C)** Effect of methanol concentration on growth (100 mM-1 M) in ES2. All experiments were performed in duplicate.

Methanol metabolism of acetogens is well-known in *A. woodii* and *E. limosum*, and the related genes were identified through transcriptomic and proteomic approaches. The genes encoding the methanol-specific methyltransferase were clustered in the form of an operon, and the mta operon consisted of eight genes (Kremp et al., 2018; Kim et al., 2021; Kremp and Müller, 2021). Accordingly, for the analysis of predicted methanol metabolic pathways, the mta gene clusters of four strains were compared. The mta operon was well conserved in the three strain genomes, except for the absence of the operon in the genome of the ES3 strain (Figure 6A).

To determine their potential for methylotrophic growth, the four strains were cultured for 1 week in a medium supplemented with 50 mM methanol. All strains, except ES3, could grow on methanol as a carbon and energy source and produced acetate as the main product. *E. limosum* showed the highest OD, while ES2 showed the highest acetate production level (Figure 6B). This might be because *E. limosum* produces various metabolites, such as butanol and acetone from methanol (Flaiz et al., 2021; Kim et al., 2021; Wood et al., 2022). To examine the effect of methanol concentrations on the growth of ES2, a growth test was performed at methanol concentrations of 0.05–1 M. ES2 showed the highest growth rate and acetate production level at methanol concentrations of 0.05–0.1 M, and its growth was significantly inhibited at 0.5 M or higher (Figure 6C). According to a recent report, *A. woodii* can grow in methanol at concentrations reaching 0.9 M (Kremp et al., 2018). Acetogens are highly resistant to methanol because it is directly assimilated into the THF system without forming the highly reactive intermediate formaldehyde (Figure 1). Furthermore, we tested whether the ES strains could grow using formaldehyde as a substrate, but they did not grow. The growth rate (0.08 h^−1^) of the ES2 strain was the highest at a methanol concentration of 50 mM (Supplementary Figure S6). The growth rate of acetogen with methanol was previously reported to be in the range of 0.07–0.1 h^−1^, and the growth rate of *E. limosum* was the highest at 0.11 h^−1^ (Pacaud et al., 1986; Cotton et al., 2020). As the utilization of methanol through the WLP theoretically requires CO_2_ as an additional carbon source, this can be an environment-friendly approach to fix CO_2_ using industrial by-products (Figure 1; Fischer et al., 2021).

### 3.6. Mixotrophic growth of ES2 strain

**Figure 7:**
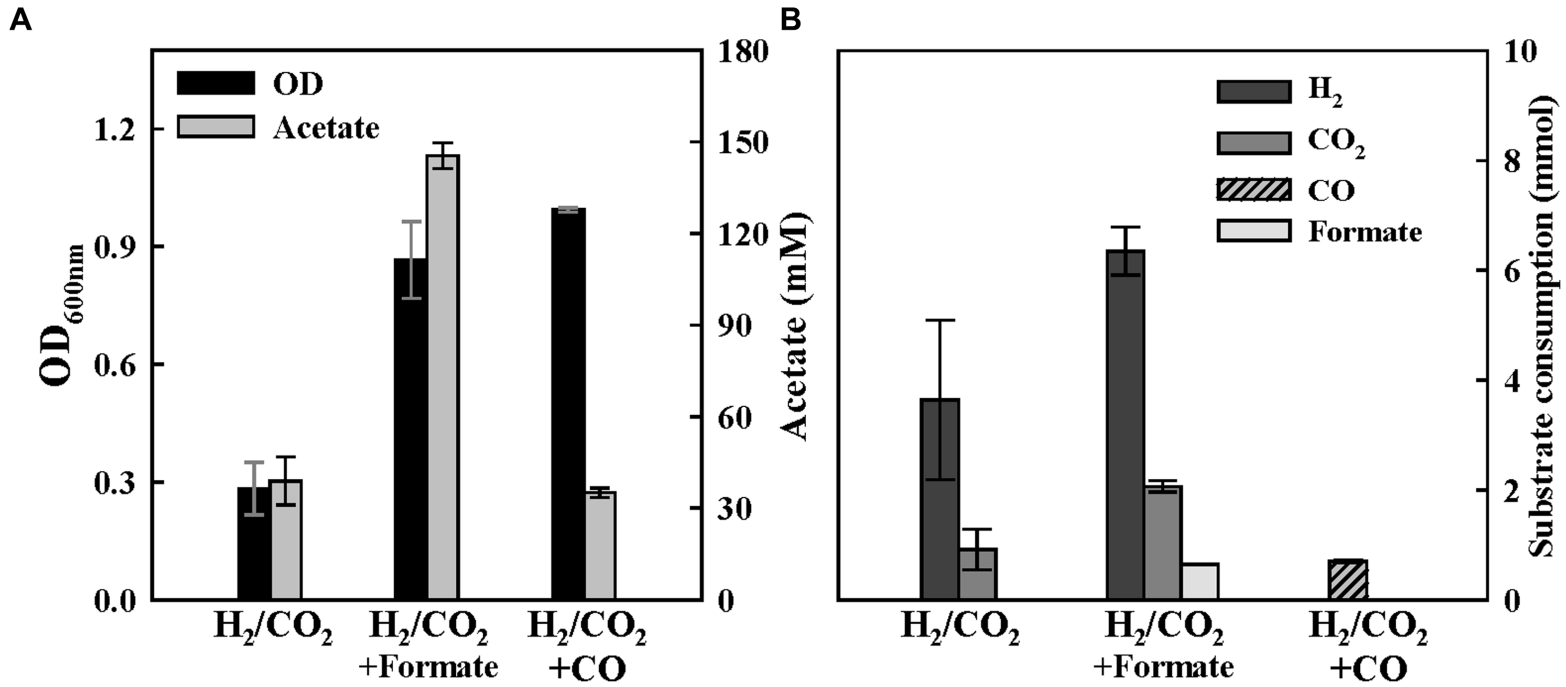
Mixotrophic growth of ES2 with additional C1 compound as an electron donor. **(A)** Final optical densities and product concentration after 1 week of batch culture. 60 mM of Formate and 15% of CO were supplemented in H_2_/CO_2_ medium. **(B)** Final substrate consumption in each condition. All experiments were performed in duplicate.

The mixotrophic growth on C1 substrates can serve as a strategy to overcome the limitations in the mass transfer of gaseous substrates and energy supply constraints, which inhibit cell growth and product spectrum. ES strains are expected to be suitable for the mixotrophic growth of various C1 combinations due to their ability to convert a wide range of C1 compounds. We tested ES strains on a culture medium supplemented with 100 mM of formate or 15% of CO, along with the presence of H_2_/CO_2_ in the headspace, and compared the final OD and product yields with those obtained under the condition where only the gaseous substrates (H_2_/CO_2_) were available. When formate was added to the medium in the presence of the gaseous substrates, we observed a significant increase in both OD and acetate production. ES2 consumed all supplemented formate and doubled the final H_2_/CO_2_ consumption (Figure 7). This result demonstrates that the mixotrophic culture can promote substrate consumption and display faster cell growth and improved productivity than single substrates. Although we supplemented the medium with 20 mM of HEPES for pH buffering, the accelerated gas consumption and increase in pH might have resulted from formate consumption. When the H_2_/CO_2_ medium was supplemented with 15% CO, the OD increased approximately thrice, but there was no significant difference in acetate production (Figure 7). Interestingly, the ES2 strain only consumed CO when both H_2_/CO_2_ and CO were present as substrates. The concentration of the remaining CO gas at the end of incubation was below 5%. These results indicate that the hydrogenase activity of ES2 strain may be sensitively inhibited in the presence of CO. However, the inhibitory effect of CO on [Fe-Fe]-hydrogenase was reversible, unlike that of O_2_ (Bennett et al., 2000; Ceccaldi et al., 2017). Therefore, if all the supplied CO gas was depleted, then H_2_/CO_2_ substrate would have also been consumed. Furthermore, as demonstrated by the growth experiments with CO, ES2 exhibits remarkable growth rates and high resistance to CO (Figures 5, 6). The mixotrophic growth of ES2 under H_2_/CO_2_ supplemented with 15% CO gas confirms its potential as a promising candidate for utilizing syngas generated as an industrial by-product.

## 4. Conclusion

In this study, we report the isolation of two new bacterial strains of the family *Eubacteriaceae* ES2 and ES3 from sediment collected at Eulsukdo, a delta island in South Korea. Both strains clustered in the coherent branch were phylogenetically located between the genera *Acetobacterium* and *Eubacterium*. By comparing the AAI index, we confirmed that ES2 and ES3 represent novel bacterial strains at the genus level. Comparative genomic analysis with phylogenetically related acetogenic bacteria *A. woodii* and *E. limosum* revealed distinct genetic characteristics in the ES strains that could potentially influence their use in C1 compound conversions. While the final metabolite observed in this study was limited to acetate, although the genomes of ES strains completely or partially encode enzymes to generate higher-compounds such as ethanol, lactate and 2,3-BDO (Supplementary Figure S8). Accordingly, ES strains possess the potential to expand their product spectrum through the optimization of culture conditions or genetic modification. In this study, we introduce novel acetogenic strains and investigated their genetic and physiological characteristics to expand their utility. In conclusion, we propose ES2 strain as a promising candidate for industrial applications in utilizing C1 compounds, given its unique features, such as high resistance and rapid growth rate on C1 substrates. These findings pave the way for future research exploring the versatile potential of ES strains in the C1-based biomanufacturing field.

## Data availability statement

The original contributions presented in the study are publicly available. This data can be found at: https://www.ncbi.nlm.nih.gov/nuccore/CP130740.1/ and https://www.ncbi.nlm.nih.gov/nuccore/CP130741.1.

## Author contributions

JY: Data curation, Formal analysis, Investigation, Methodology, Resources, Writing – original draft. M-JP: Data curation, Formal analysis, Writing – original draft. JL: Data curation, Formal analysis, Writing – original draft. SJK: Formal analysis, Methodology, Writing – review & editing. JKL: Data curation, Formal analysis, Writing – original draft. HSL: Conceptualization, Supervision, Validation, Writing – review & editing. SGK: Conceptualization, Supervision, Validation, Writing – review & editing. J-HL: Funding acquisition, Supervision, Writing – review & editing. KKK: Funding acquisition, Supervision, Writing – review & editing. YJK: Conceptualization, Investigation, Project administration, Writing – review & editing.

## Footnotes

^1^ https://orthovenn2.bioinfotoolkits.net

## References

[ref1] Abrini J. Naveau H. Nyns E.-J. (1994). Clostridium autoethanogenum, sp. nov., an anaerobic bacterium that produces ethanol from carbon monoxide. Arch. Microbiol. 161, 345–351. doi: 10.1007/BF00303591

[ref2] Andreesen J. R. Ljungdahl L. G. (1973). Formate dehydrogenase of Clostridium thermoaceticum: incorporation of selenium-75, and the effects of selenite, molybdate, and tungstate on the enzyme. J. Bacteriol. 116, 867–873. doi: 10.1128/jb.116.2.867-873.1973, PMID: 4147651 PMC285457

[ref3] Asnicar F. Thomas A. M. Beghini F. Mengoni C. Manara S. Manghi P.. (2020). Precise phylogenetic analysis of microbial isolates and genomes from metagenomes using PhyloPhlAn 3.0. Nat. Commun. 11:2500. doi: 10.1038/s41467-020-16366-7, PMID: 32427907 PMC7237447

[ref4] Bennett B. Lemon B. J. Peters J. W. (2000). Reversible carbon monoxide binding and inhibition at the active site of the Fe-only hydrogenase. Biochemistry 39, 7455–7460. doi: 10.1021/bi992583z, PMID: 10858294

[ref5] Bertsch J. Müller V. (2015). CO metabolism in the acetogen *Acetobacterium woodii*. Appl. Environ. Microbiol. 81, 5949–5956. doi: 10.1128/AEM.01772-15, PMID: 26092462 PMC4551271

[ref6] Burger Y. Schwarz F. M. Müller V. (2022). Formate-driven H_2_ production by whole cells of *Thermoanaerobacter kivui*. Biotechnol. Biofuels Bioproducts 15:48. doi: 10.1186/s13068-022-02147-5, PMID: 35545791 PMC9097184

[ref7] Ceccaldi P. Schuchmann K. Müller V. Elliott S. J. (2017). The hydrogen dependent CO_2_ reductase: the first completely CO tolerant FeFe-hydrogenase. Energy Environ. Sci. 10, 503–508. doi: 10.1039/C6EE02494G

[ref25] Chang I.-S. Kim D.-H. Kim B.-H. Shin P.-K. Sung H.-C. Lovitt R.W. (1998). CO fermentation of Eubacterium limosum KIST612. Journal of Microbiology and Biotechnology 8, 134–140.

[ref8] Chen J. Yang S. Qian Y. (2019). A novel path for carbon-rich resource utilization with lower emission and higher efficiency: an integrated process of coal gasification and coking to methanol production. Energy 177, 304–318. doi: 10.1016/j.energy.2019.03.161

[ref9] Claassens N. J. Cotton C. A. Kopljar D. Bar-Even A. (2019). Making quantitative sense of electromicrobial production. Nature Catalysis 2, 437–447. doi: 10.1038/s41929-019-0272-0

[ref10] Cotton C. A. Claassens N. J. Benito-Vaquerizo S. Bar-Even A. (2020). Renewable methanol and formate as microbial feedstocks. Curr. Opin. Biotechnol. 62, 168–180. doi: 10.1016/j.copbio.2019.10.002, PMID: 31733545

[ref11] Deaton J. C. Solomon E. I. Watt G. D. Wetherbee P. J. Durfor C. N. (1987). Electron paramagnetic resonance studies of the tungsten-containing formate dehydrogenase from Clostridium thermoaceticum. Biochem. Biophys. Res. Commun. 149, 424–430. doi: 10.1016/0006-291X(87)90384-6 2827642

[ref12] Denned A. (2006). Acetogenic prokaryotes. Prokaryotes 2, 354–420. doi: 10.1007/0-387-30742-7_13

[ref13] Dietrich H. M. Kremp F. Öppinger C. Ribaric L. Müller V. (2021). Biochemistry of methanol-dependent acetogenesis in *Eubacterium callanderi* KIST612. Environ. Microbiol. 23, 4505–4517. doi: 10.1111/1462-2920.15643, PMID: 34125457

[ref14] Drake H. L. Gößner A. S. Daniel S. L. (2008). Old acetogens, new light. Ann. N. Y. Acad. Sci. 1125, 100–128. doi: 10.1196/annals.1419.016, PMID: 18378590

[ref15] Esposito A. Tamburini S. Triboli L. Ambrosino L. Chiusano M. L. Jousson O. (2019). Insights into the genome structure of four acetogenic bacteria with specific reference to the Wood-Ljungdahl pathway. Microbiology 8:e938. doi: 10.1002/mbo3.938, PMID: 31573151 PMC6925170

[ref16] Felsenstein J. (1981). Evolutionary trees from DNA sequences: a maximum likelihood approach. J. Mol. Evol. 17, 368–376. doi: 10.1007/BF01734359 7288891

[ref17] Fischer P. Q. Sánchez-Andrea I. Stams A. J. Villanueva L. Sousa D. Z. (2021). Anaerobic microbial methanol conversion in marine sediments. Environ. Microbiol. 23, 1348–1362. doi: 10.1111/1462-2920.15434, PMID: 33587796 PMC8048578

[ref18] Fitch W. M. (1971). Toward defining the course of evolution: minimum change for a specific tree topology. Syst. Biol. 20, 406–416. doi: 10.1093/sysbio/20.4.406

[ref19] Flaiz M. Ludwig G. Bengelsdorf F. R. Dürre P. (2021). Production of the biocommodities butanol and acetone from methanol with fluorescent FAST-tagged proteins using metabolically engineered strains of *Eubacterium limosum*. Biotechnol. Biofuels 14:117. doi: 10.1186/s13068-021-01966-2, PMID: 33971948 PMC8111989

[ref20] Grunwald S. Mottet A. Grousseau E. Plassmeier J. K. Popović M. K. Uribelarrea J. L.. (2015). Kinetic and stoichiometric characterization of organoautotrophic growth of *Ralstonia eutropha* on formic acid in fed-batch and continuous cultures. Microb. Biotechnol. 8, 155–163. doi: 10.1111/1751-7915.12149, PMID: 25123319 PMC4321381

[ref21] Hartmann T. Schwanhold N. Leimkühler S. (2015). Assembly and catalysis of molybdenum or tungsten-containing formate dehydrogenases from bacteria. Biochimica et Biophysica Acta (BBA)-Proteins and Proteomics 1854, 1090–1100. doi: 10.1016/j.bbapap.2014.12.006 25514355

[ref22] Jukes T. H. Cantor C. R. (1969). “Evolution of protein molecules” in Mammalian protein metabolism. ed. Munro H. N. (New York: Academic Press), 21–132.

[ref23] Kang S. Song Y. Jin S. Shin J. Bae J. Kim D. R.. (2020). Adaptive laboratory evolution of *Eubacterium limosum* ATCC 8486 on carbon monoxide. Front. Microbiol. 11:402. doi: 10.3389/fmicb.2020.00402, PMID: 32218779 PMC7079680

[ref24] Katoh K. Standley D. M. (2013). MAFFT multiple sequence alignment software version 7: improvements in performance and usability. Mol. Biol. Evol. 30, 772–780. doi: 10.1093/molbev/mst010, PMID: 23329690 PMC3603318

[ref26] Kim J.-Y. Park S. Jeong J. Lee M. Kang B. Jang S. H.. (2021). Methanol supply speeds up synthesis gas fermentation by methylotrophic-acetogenic bacterium, *Eubacterium limosum* KIST612. Bioresour. Technol. 321:124521. doi: 10.1016/j.biortech.2020.124521, PMID: 33321298

[ref27] Köpke M. Mihalcea C. Bromley J. C. Simpson S. D. (2011). Fermentative production of ethanol from carbon monoxide. Curr. Opin. Biotechnol. 22, 320–325. doi: 10.1016/j.copbio.2011.01.005 21353524

[ref28] Kottenhahn P. Schuchmann K. Müller V. (2018). Efficient whole cell biocatalyst for formate-based hydrogen production. Biotechnol. Biofuels 11, 93–99. doi: 10.1186/s13068-018-1082-3, PMID: 29619089 PMC5879573

[ref29] Kremp F. Müller V. (2021). Methanol and methyl group conversion in acetogenic bacteria: biochemistry, physiology and application. FEMS Microbiol. Rev. 45:fuaa040. doi: 10.1093/femsre/fuaa040, PMID: 32901799

[ref30] Kremp F. Poehlein A. Daniel R. Müller V. (2018). Methanol metabolism in the acetogenic bacterium *Acetobacterium woodii*. Environ. Microbiol. 20, 4369–4384. doi: 10.1111/1462-2920.14356, PMID: 30003650

[ref31] Kumar S. Stecher G. Tamura K. (2016). MEGA7: molecular evolutionary genetics analysis version 7.0 for bigger datasets. Mol. Biol. Evol. 33, 1870–1874. doi: 10.1093/molbev/msw054, PMID: 27004904 PMC8210823

[ref32] Lee H. Bae J. Jin S. Kang S. Cho B.-K. (2022). Engineering acetogenic bacteria for efficient one-carbon utilization. Front. Microbiol. 13:865168. doi: 10.3389/fmicb.2022.865168, PMID: 35615514 PMC9124964

[ref33] Lee J. Lee J. W. Chae C. G. Kwon S. J. Kim Y. J. Lee J.-H.. (2019). Domestication of the novel alcohologenic acetogen Clostridium sp. AWRP: from isolation to characterization for syngas fermentation. Biotechnol. Biofuels 12, 1–14. doi: 10.1186/s13068-019-1570-0 31572495 PMC6757427

[ref34] Li X. Luo Q. Wofford N. Q. Keller K. L. Mcinerney M. J. Wall J. D.. (2009). A molybdopterin oxidoreductase is involved in H_2_ oxidation in *Desulfovibrio desulfuricans* G20. J. Bacteriol. 191, 2675–2682. doi: 10.1128/jb.01814-08, PMID: 19233927 PMC2668429

[ref35] Liew F. Martin M. E. Tappel R. C. Heijstra B. D. Mihalcea C. Köpke M. (2016). Gas fermentation—a flexible platform for commercial scale production of low-carbon-fuels and chemicals from waste and renewable feedstocks. Front. Microbiol. 7:694. doi: 10.3389/fmicb.2016.00694, PMID: 27242719 PMC4862988

[ref36] Liu C. Mortenson L. (1984). Formate dehydrogenase of *Clostridium pasteurianum*. J. Bacteriol. 159, 375–380. doi: 10.1128/jb.159.1.375-380.1984, PMID: 6547435 PMC215640

[ref37] Lv X. Yu W. Zhang C. Ning P. Li J. Liu Y.. (2022). C1-based biomanufacturing: advances, challenges and perspectives. Bioresour. Technol. 367:128259. doi: 10.1016/j.biortech.2022.128259, PMID: 36347475

[ref38] Marcellin E. Behrendorff J. B. Nagaraju S. Detissera S. Segovia S. Palfreyman R. W.. (2016). Low carbon fuels and commodity chemicals from waste gases–systematic approach to understand energy metabolism in a model acetogen. Green Chem. 18, 3020–3028. doi: 10.1039/C5GC02708J

[ref39] Meier-Kolthoff J. P. Auch A. F. Klenk H.-P. Göker M. (2013). Genome sequence-based species delimitation with confidence intervals and improved distance functions. BMC Bioinformat 14, 1–14. doi: 10.1186/1471-2105-14-60, PMID: 23432962 PMC3665452

[ref40] Moon J. Dönig J. Kramer S. Poehlein A. Daniel R. Müller V. (2021). Formate metabolism in the acetogenic bacterium *Acetobacterium woodii*. Environ. Microbiol. 23, 4214–4227. doi: 10.1111/1462-2920.15598, PMID: 33989450

[ref41] Moon J. Jain S. Müller V. Basen M. (2020). Homoacetogenic conversion of mannitol by the thermophilic acetogenic bacterium *Thermoanaerobacter kivui* requires external CO_2_. Front. Microbiol. 11:571736. doi: 10.3389/fmicb.2020.571736, PMID: 33042077 PMC7522397

[ref42] Müller V. (2003). Energy conservation in acetogenic bacteria. Appl. Environ. Microbiol. 69, 6345–6353. doi: 10.1128/AEM.69.11.6345-6353.2003, PMID: 14602585 PMC262307

[ref43] Müller V. (2019). New horizons in acetogenic conversion of one-carbon substrates and biological hydrogen storage. Trends Biotechnol. 37, 1344–1354. doi: 10.1016/j.tibtech.2019.05.008, PMID: 31257058

[ref44] Pacaud S. Loubiere P. Goma G. Lindley N. (1986). Effects of various organic acid supplements on growth rates of *Eubacterium limosum* B2 on methanol. Appl. Microbiol. Biotechnol. 24, 75–78. doi: 10.1007/BF00266289

[ref45] Price M. N. Dehal P. S. Arkin A. P. (2009). FastTree: computing large minimum evolution trees with profiles instead of a distance matrix. Mol. Biol. Evol. 26, 1641–1650. doi: 10.1093/molbev/msp077, PMID: 19377059 PMC2693737

[ref46] Qin Q.-L. Xie B.-B. Zhang X.-Y. Chen X.-L. Zhou B.-C. Zhou J.. (2014). A proposed genus boundary for the prokaryotes based on genomic insights. J. Bacteriol. 196, 2210–2215. doi: 10.1128/jb.01688-14, PMID: 24706738 PMC4054180

[ref47] Ross D. E. Marshall C. W. Gulliver D. May H. D. Norman R. S. (2020). Defining genomic and predicted metabolic features of the Acetobacterium genus. Msystems 5, e00277–e00220. doi: 10.1128/msystems.00277-20 32934112 PMC7498680

[ref48] Saitou N. Nei M. (1987). The neighbor-joining method: a new method for reconstructing phylogenetic trees. Mol. Biol. Evol. 4, 406–425. doi: 10.1093/oxfordjournals.molbev.a040454, PMID: 3447015

[ref49] Sattley W. M. Madigan M. T. (2007). Cold-active acetogenic bacteria from surficial sediments of perennially ice-covered Lake Fryxell, Antarctica. FEMS Microbiol. Lett. 272, 48–54. doi: 10.1111/j.1574-6968.2007.00737.x, PMID: 17456187

[ref50] Schuchmann K. Müller V. (2013). Direct and reversible hydrogenation of CO_2_ to formate by a bacterial carbon dioxide reductase. Science 342, 1382–1385. doi: 10.1126/science.1244758, PMID: 24337298

[ref51] Schuchmann K. Müller V. (2014). Autotrophy at the thermodynamic limit of life: a model for energy conservation in acetogenic bacteria. Nat. Rev. Microbiol. 12, 809–821. doi: 10.1038/nrmicro3365, PMID: 25383604

[ref52] Schuchmann K. Vonck J. Müller V. (2016). A bacterial hydrogen-dependent CO_2_ reductase forms filamentous structures. FEBS J. 283, 1311–1322. doi: 10.1111/febs.13670, PMID: 26833643

[ref53] Schwarz F. M. Schuchmann K. Müller V. (2018). Hydrogenation of CO_2_ at ambient pressure catalyzed by a highly active thermostable biocatalyst. Biotechnol. Biofuels 11, 237–211. doi: 10.1186/s13068-018-1236-3, PMID: 30186365 PMC6119302

[ref54] Seemann T. (2014). Prokka: rapid prokaryotic genome annotation. Bioinformatics 30, 2068–2069. doi: 10.1093/bioinformatics/btu153, PMID: 24642063

[ref55] Song Y. Shin J. Jeong Y. Jin S. Lee J.-K. Kim D. R.. (2017). Determination of the genome and primary transcriptome of syngas fermenting *Eubacterium limosum* ATCC 8486. Sci. Rep. 7:13694. doi: 10.1038/s41598-017-14123-3, PMID: 29057933 PMC5651825

[ref56] Stamatakis A. (2014). RAxML version 8: a tool for phylogenetic analysis and post-analysis of large phylogenies. Bioinformatics 30, 1312–1313. doi: 10.1093/bioinformatics/btu033, PMID: 24451623 PMC3998144

[ref57] Tamura K. Nei M. Kumar S. (2004). Prospects for inferring very large phylogenies by using the neighbor-joining method. Proc. Natl. Acad. Sci. 101, 11030–11035. doi: 10.1073/pnas.040420610, PMID: 15258291 PMC491989

[ref58] Tanner R. S. Miller L. M. Yang D. (1993). *Clostridium ljungdahlii* sp. nov., an acetogenic species in clostridial rRNA homology group I. Int. J. Syst. Bacteriol. 43, 232–236. doi: 10.1099/00207713-43-2-232, PMID: 7684239

[ref59] Tremblay P.-L. Höglund D. Koza A. Bonde I. Zhang T. (2015). Adaptation of the autotrophic acetogen *Sporomusa ovata* to methanol accelerates the conversion of CO_2_ to organic products. Sci. Rep. 5, 1–11. doi: 10.1038/srep16168, PMID: 26530351 PMC4632017

[ref60] Van Dongen S. M. (2000). Graph clustering by flow simulation. Doctoral dissertation. Utrecht: University of Utrecht.

[ref61] Wang S. Huang H. Kahnt J. Mueller A. P. Köpke M. Thauer R. K. (2013). NADP-specific electron-bifurcating [FeFe]-hydrogenase in a functional complex with formate dehydrogenase in Clostridium autoethanogenum grown on CO. J. Bacteriol. 195, 4373–4386. doi: 10.1128/jb.00678-13, PMID: 23893107 PMC3807470

[ref62] Weghoff M. C. Müller V. (2016). CO metabolism in the thermophilic acetogen *Thermoanaerobacter kivui*. Appl. Environ. Microbiol. 82, 2312–2319. doi: 10.1128/AEM.00122-16, PMID: 26850300 PMC4959504

[ref63] Wiechmann A. Müller V. (2019). “Synthesis of acetyl-CoA from carbon dioxide in acetogenic bacteria” in Biogenesis of fatty acids, lipids and membranes, handbook of hydrocarbon and lipid microbiology. ed. Geiger O. (Cham: Springer), 1–18.

[ref64] Wiesberg I. L. Brigagão G. V. Ofélia De Queiroz F. A. De Medeiros J. L. (2019). Carbon dioxide management via exergy-based sustainability assessment: carbon capture and storage versus conversion to methanol. Renew. Sust. Energ. Rev. 112, 720–732. doi: 10.1016/j.rser.2019.06.032

[ref65] Wood J. C. Gonzalez-Garcia R. A. Daygon D. Talbo G. Plan M. R. Marcellin E.. (2022). Characterisation of acetogen formatotrophic potential using *E. limosum*. bio Rxiv. doi: 10.1101/2022.11.02.514939 37272938

[ref66] Yamamoto I. Saiki T. Liu S.-M. Ljungdahl L. G. (1983). Purification and properties of NADP-dependent formate dehydrogenase from Clostridium thermoaceticum, a tungsten-selenium-iron protein. J. Biol. Chem. 258, 1826–1832. doi: 10.1016/S0021-9258(18)33062-X, PMID: 6822536

[ref67] Yasin M. Jeong Y. Park S. Jeong J. Lee E. Y. Lovitt R. W.. (2015). Microbial synthesis gas utilization and ways to resolve kinetic and mass-transfer limitations. Bioresour. Technol. 177, 361–374. doi: 10.1016/j.biortech.2014.11.022, PMID: 25443672

[ref68] Yishai O. Lindner S. N. De La Cruz J. G. Tenenboim H. Bar-Even A. (2016). The formate bio-economy. Curr. Opin. Chem. Biol. 35, 1–9. doi: 10.1016/j.cbpa.2016.07.005, PMID: 27459678

[ref69] Yoon S.-H. Ha S.-M. Lim J. Kwon S. Chun J. (2017). A large-scale evaluation of algorithms to calculate average nucleotide identity. Antonie Van Leeuwenhoek 110, 1281–1286. doi: 10.1007/s10482-017-0844-4 28204908

