# Supplementary material for: Genomic potential and physiological characteristics of C1 metabolism in novel acetogenic bacteria
Source: Front Microbiol. 2023 Oct 19;14:1279544. doi: 10.3389/fmicb.2023.1279544 (PMC10625859; doi:10.3389/fmicb.2023.1279544)
Supplement: Supplementary file 1 [file Data_Sheet_1.pdf]

Supplementary Material

Genomic potential and physiological characteristics of C1 metabolism in novel acetogenic bacteria

Jihyun Yu[1,2], Mi-Jeong Park[1,2], Joungmin Lee[1], Soo Jae Kwon[1,2], Jae Kyu Lim[1,2], Hyun Sook Lee[1,2], Sung Gyun Kang[1,2], Jung-Hyun Lee[1,2], Kae Kyoung Kwon[1,2], and Yun Jae Kim[1,2*]

[1]Korea Institute of Ocean Science and Technology, Busan 49111, Republic of Korea
[2]KIOST School, University of Science and Technology, Daejeon 34113, Republic of Korea

***Correspondence:**
Y.J. Kim
bio1213@kiost.ac.kr

Supplementary Tables

Table S1. Optimal growth conditions of ES and reference strains

Strains: *Acetobacterium woodii* DSM 1030^T (Sharak Genthner and Bryant, 1987; Bertsch and Müller, 2015), *Eubacterium limosum* ATCC 8486^T (Genthner and Bryant, 1982; Sharak Genthner and Bryant, 1987).

**Strain**	**ES2**	**ES3**	***A. woodii***^{T}	***E. limosum***^{T}
**Optimal NaCl %**	0.5 (0~4.5)	2.5(0~5)	0	0.5
**Optimal pH**	7.8 (5.5~8.8)	6.8 (5.5~8.8)	6.8	7.0-7.2
**Optimal temprature °C**	35 (25~40)	30 (22~42)	30 (20~30)	37-39 (30~45)

Table S2. The general genomic features of *A. woodii*, *E. limosum*, and ES strains.

Characteristic	ES2	ES3	*Acetobacterium woodii* DSM 1030^T	*Eubacterium limosum* ATCC 8486^T
**Sequencing method**	PacBio_20K	PacBio_20K	Unknown	PacBio
**Method coverage**	87.9	245.5	Unknown	200
**Assembler**	PacBio SMRT 2.3.0	PacBio SMRT 2.3.0	Unknown	PacBio SMRT 2.3.0
**Contig number**	1	1	1	1
**Genome size (Mb)**	3.76	3.39	4.04	4.42
**N50 (kbps)**	3763	3392	4044	4422
**Protein-coding genes**	3619	3046	3564	4067
**Ribosomal RNA**	16	16	16	16
**Transfer RNA**	56	59	60	61
**ncRNAs**	ND	ND	4	7
**Pseudogenes**	ND	ND	60	50
**G+C ratio (mol%)**	40.7	39.6	39.3	47.2
**GenBank Accession No.**	CP130740.1	CP130741.1	CP002987.1	CP019962.1

ND, not determined

Supplementary Figures

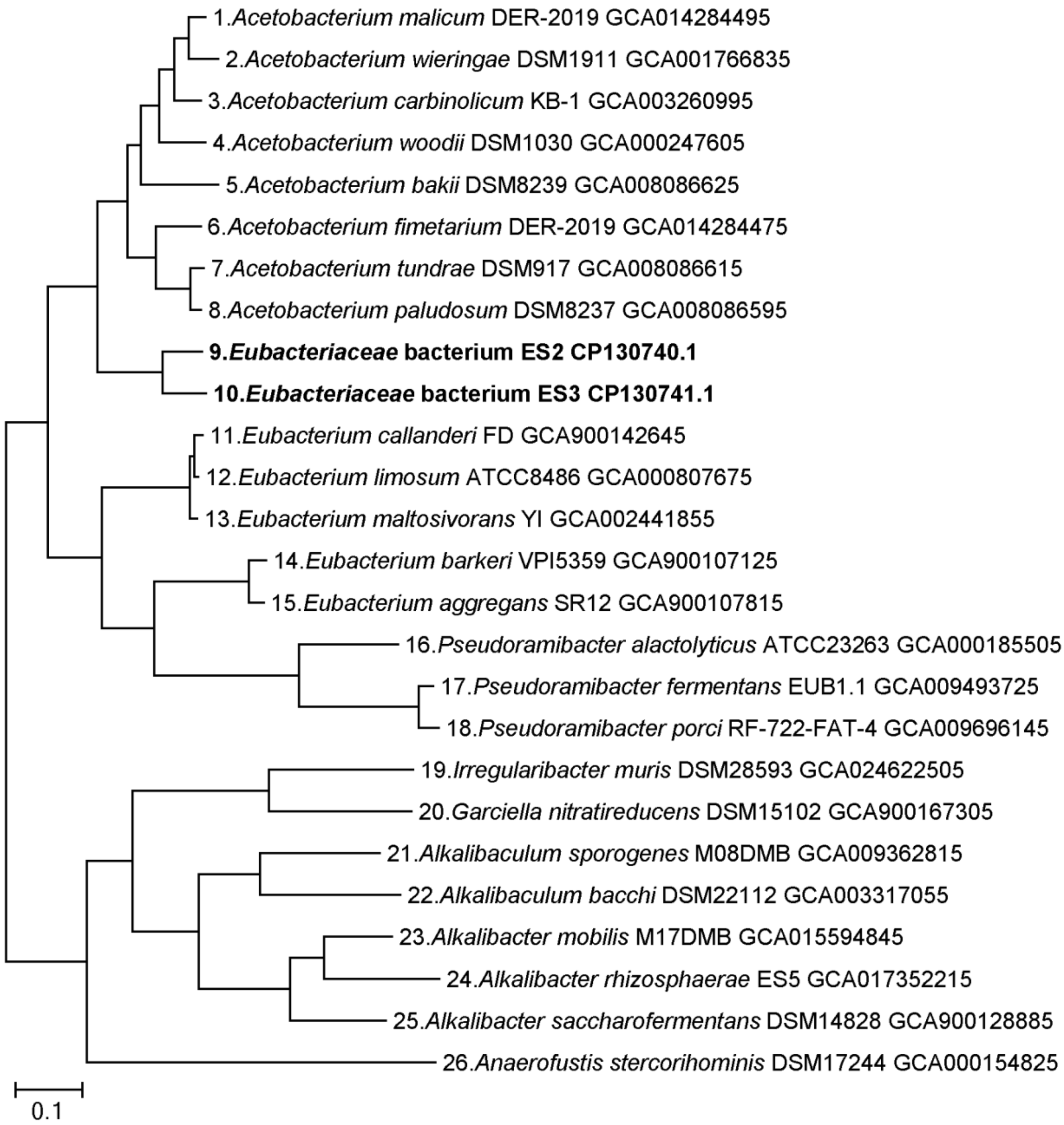

Figure S1. Phylogenomic tree of species belonging to *Eubacteriaceae*, closely related to ES2 and ES3. Utilizing 400 core gene markers, a comprehensive phylogenomic analysis was undertaken on selected strains from the *Eubacteriaceae* family. The scale bar on the tree signifies an approximate sequence divergence of 5%.

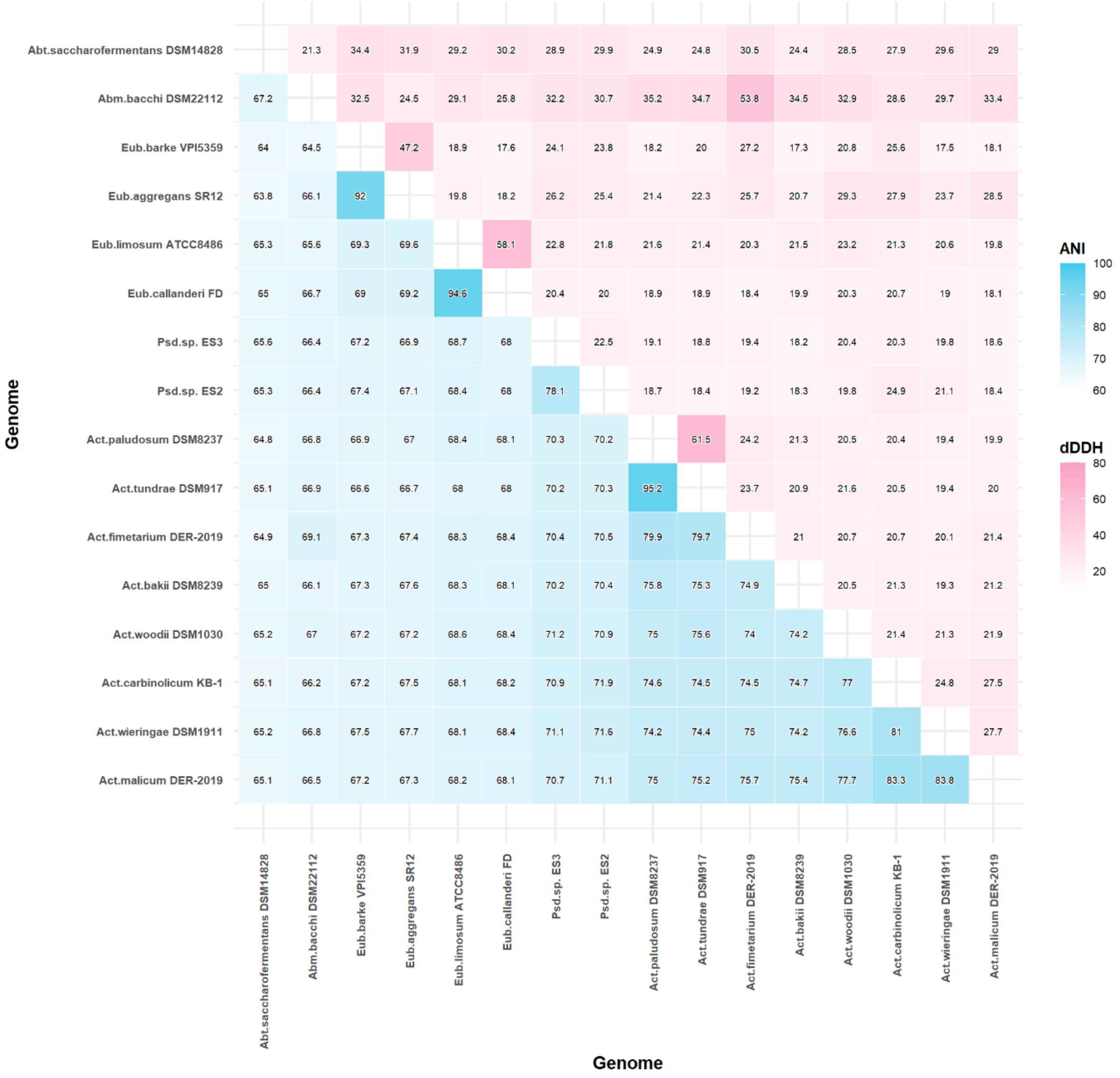

Figure S2. Genomic distance analysis on ES strains with neighboring species; Heatmap of average nucleotide identity (ANI) and *digital* DNA-DNA Hybridization (*d*DDH).

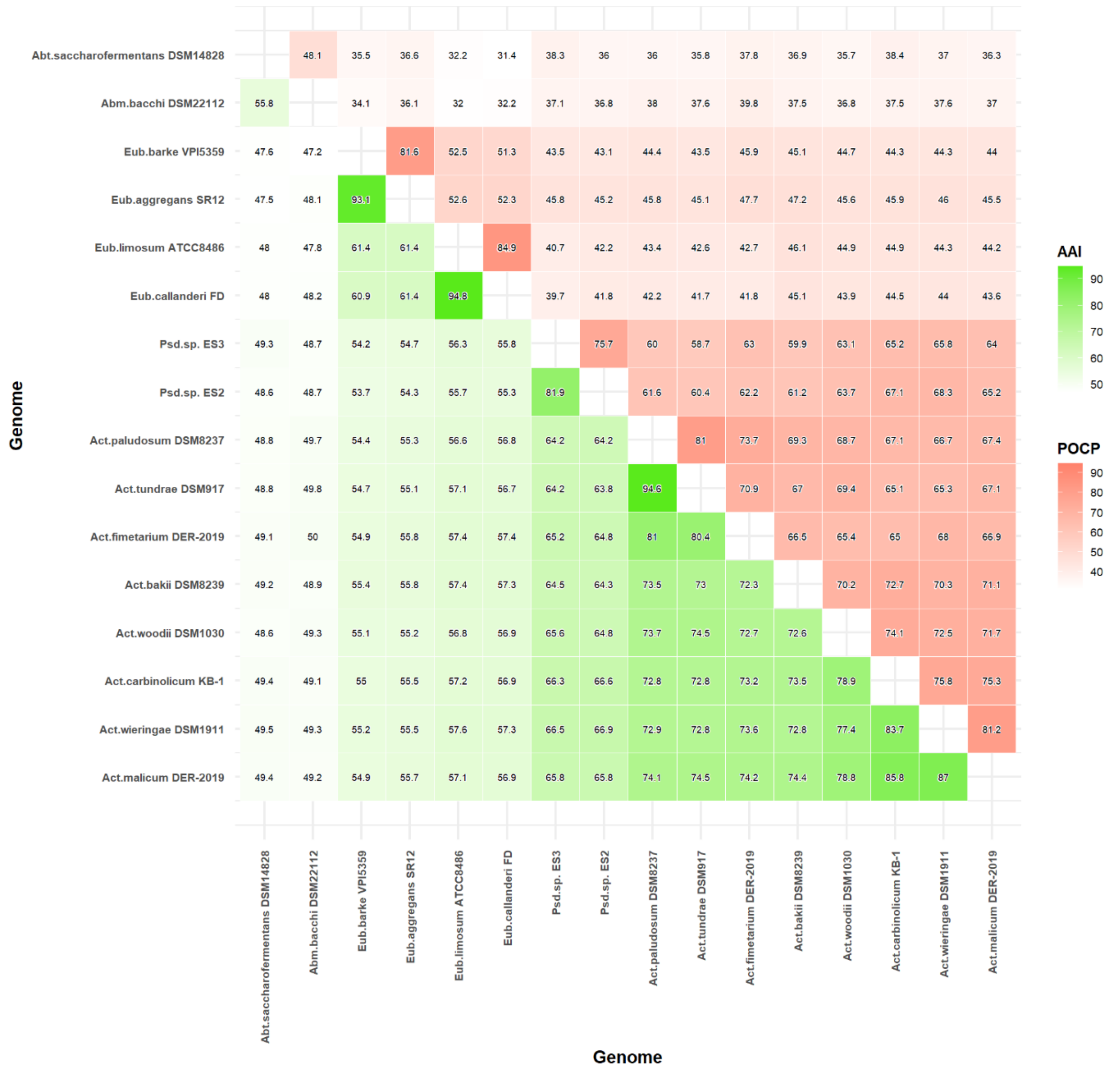

Figure S3. Genomic distance analysis on ES strains with neighboring species; Heatmap of average amino acid identity (AAI) and percentage of conserved proteins (POCP).

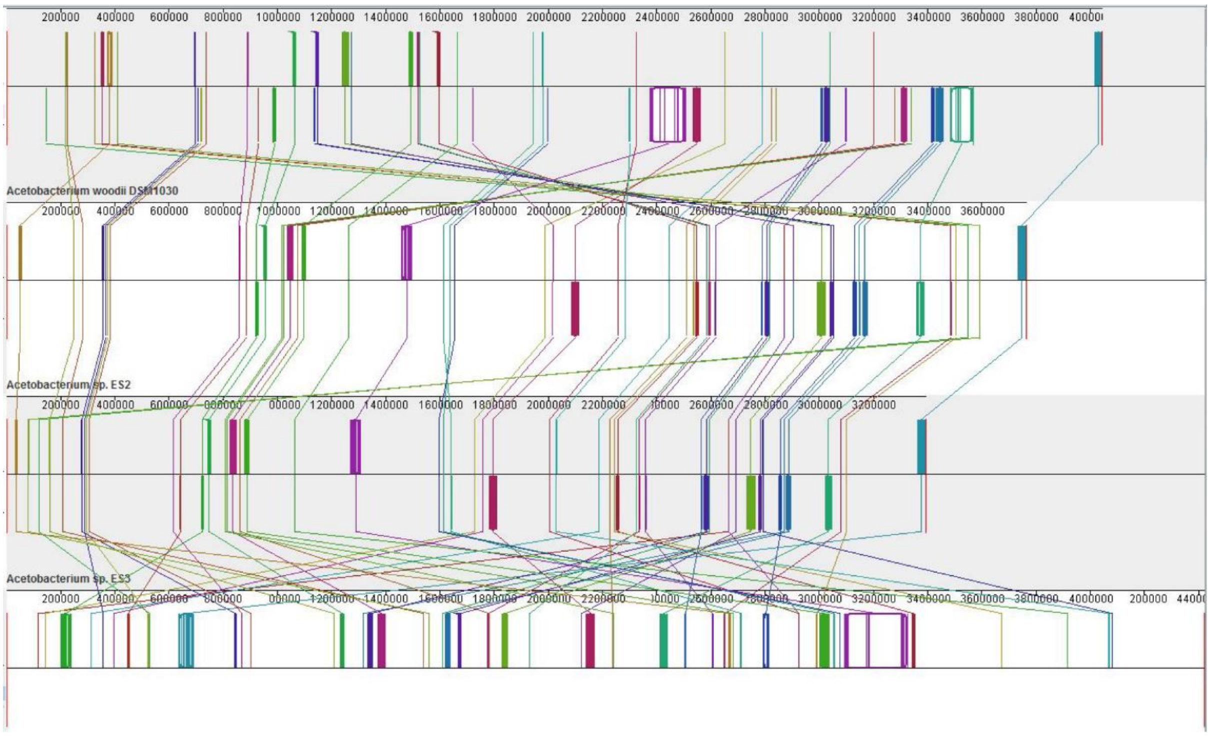

Figure S4. Synteny analysis of four strain genomes.

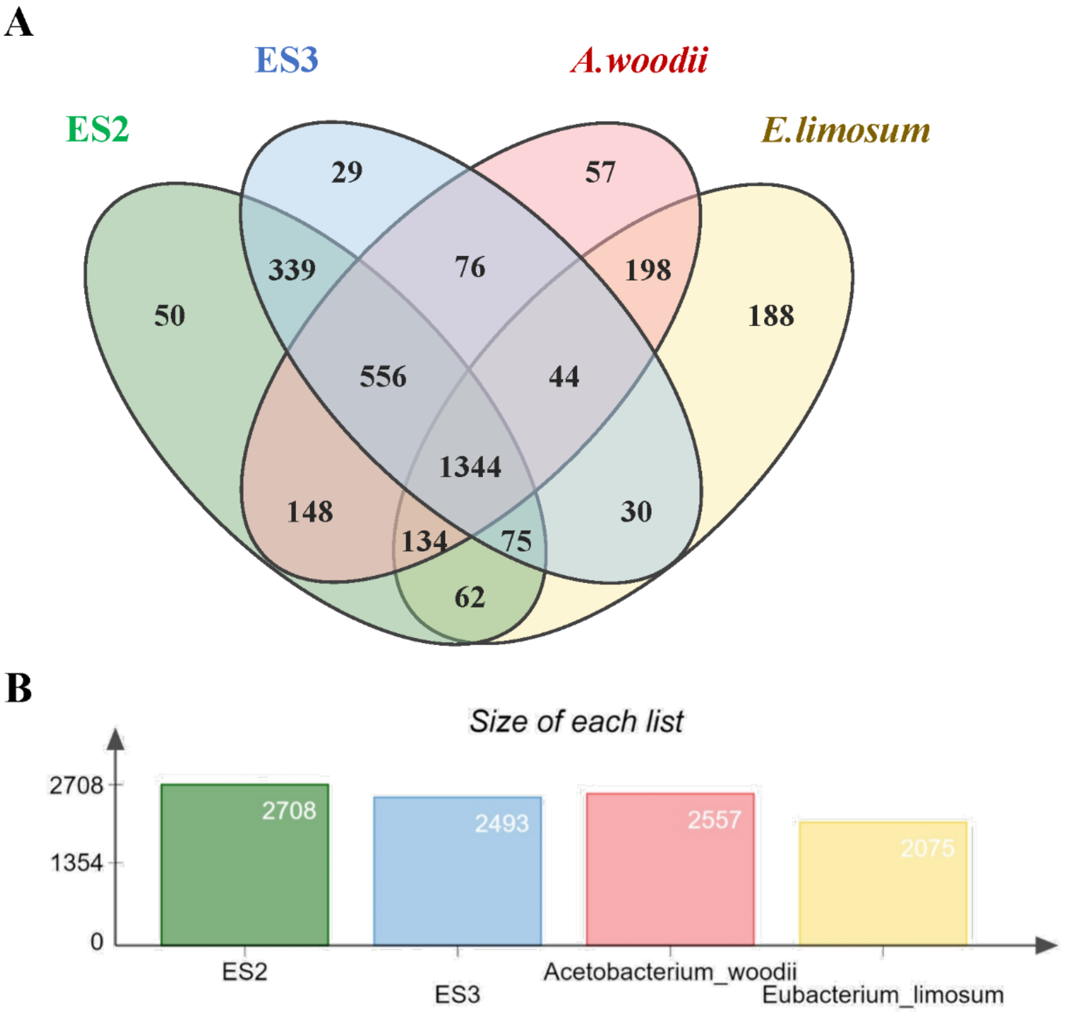

Figure S5. Orthologous gene clusters between ES strains with *A. woodii* and *E. limosum* were analyzed. (A) Venn diagram of overlapping and unique clusters in four strains. (B) Number of protein clusters found in four strains.

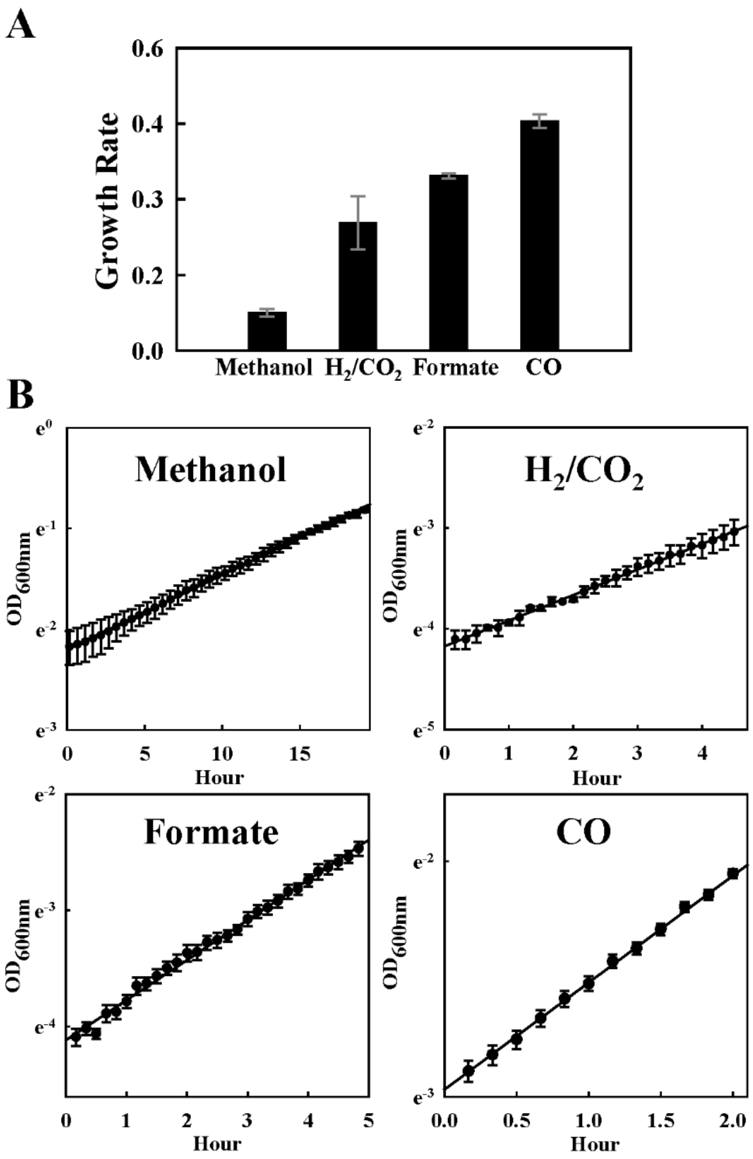

Figure S6. The growth rate of ES2 on C1 compounds. (A) growth rates were measured under four kinds of C1 compounds: 50 mM of methanol, H_2/CO_2 (8:2, 100 kPa), 200 mM of sodium formate, and 50% of CO ($CO/CO_2/H_2/N_2$, 5:1:1:3, 100 kPa). (B) The growth rate was determined by the increase in optical density (O.D.) during the exponential phase for each C1 compound. O.D. were taken at 600 nm, every ten minutes. All experiments were performed in duplicate.

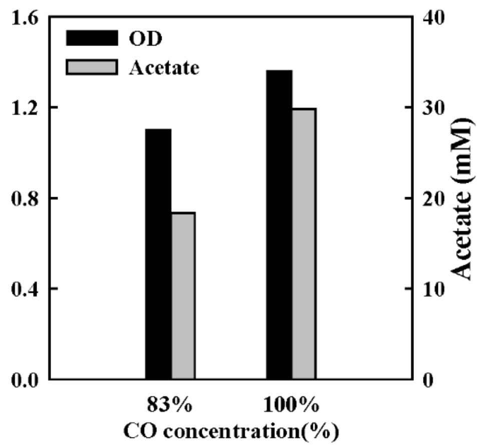

Figure S7. Growth of ES2 after adapting to a high concentration of CO gas. Final optical densities and product concentration were measured after 2 weeks of batch culture under 83% (17% of N2) and 100% of CO. Seed culture was prepared under 50% of CO.

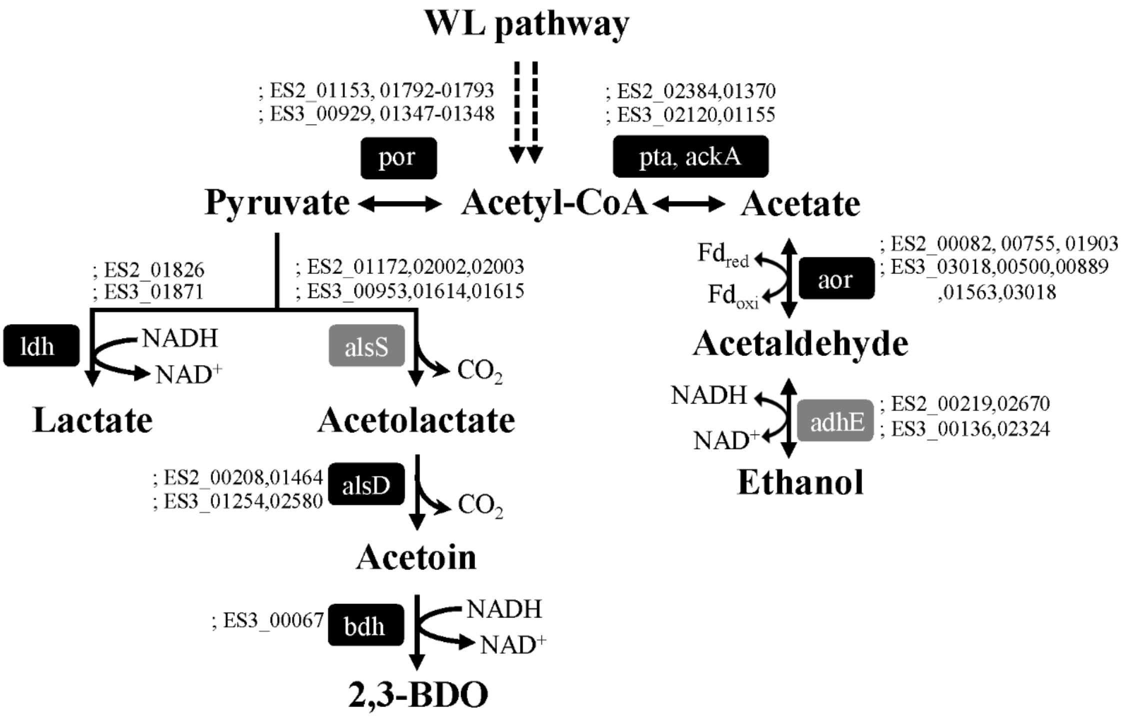

Figure S8. Pathways for the biosynthesis of lactate, ethanol, and 2,3-BDO, and the genetic potential predicted in ES strains for their production. The black and gray boxes represent the genes coding for enzymes required for the specific reactions. In particular, the black box represents a gene present in the genome of the ES strain, and the gray box represents the absence of the gene. Each locus tag indicates the location of the corresponding gene or a gene with analogous functions in the genome of the ES strains.

References

Bertsch, J., and Müller, V. (2015). CO metabolism in the acetogen *Acetobacterium woodii*. *Applied and environmental microbiology* 81**,** 5949-5956. doi: 10.1128/AEM.01772-15

Genthner, B.S., and Bryant, M. (1982). Growth of *Eubacterium limosum* with carbon monoxide as the energy source. *Applied and Environmental Microbiology* 43**,** 70-74. doi: 10.1128/aem.43.1.70-74.1982

Sharak Genthner, B., and Bryant, M. (1987). Additional characteristics of one-carbon-compound utilization by *Eubacterium limosum* and *Acetobacterium woodii*. *Applied and Environmental Microbiology* 53**,** 471-476. doi: 10.1128/aem.53.3.471-476.1987
